# How Do Females Persist? Life History, Environment, and Female Advantage in Gynodioecious *Cirsium*

**DOI:** 10.1002/ece3.74093

**Published:** 2026-07-24

**Authors:** Ashini Dias Mahadura, Jakub Šmerda, Tereza Maňáková, Michaela Kubová, Zuzana Halamová, František Zedek, Petr Bureš

**Affiliations:** ^1^ Department of Botany and Zoology, Faculty of Science Masaryk University Brno Czech Republic

**Keywords:** achene predation (granivory), *Cirsium*, environmental predictors, female advantage, gynodioecy, life history

## Abstract

Gynodioecy—the coexistence of females and hermaphrodites—raises a fundamental question in evolutionary ecology: how do females persist when hermaphrodites can both sire and set seed? We addressed this by quantifying how life‐history strategy (polycarpic vs. monocarpic), trait‐specific components of female advantage, and environmental context shape female frequencies in two gynodioecious thistles with contrasting life histories: the polycarpic *Cirsium heterophyllum* and the monocarpic *C. palustre* . Female frequencies differed sharply between species (mean 53.3% vs. 19.3%), and this divergence closely matched species‐level female advantage, calculated as the ratio of viable achene production in females relative to hermaphrodites, which was strong in *C. heterophyllum* (3.77) but weak in *C. palustre* (1.26). Across species, female advantage arose from reproductive rather than vegetative traits: females produced more florets per plant, had higher achene viability, and experienced lower predispersal granivory than hermaphrodites. However, the dominant trait component differed with life history: achene viability was the primary contributor to female advantage in polycarpic *C. heterophyllum* , whereas enhanced floret production contributed most strongly in monocarpic *C. palustre* . Environmental predictors had limited explanatory power, with temperature seasonality (BIO4) accounting for only a small fraction (6.5%) of variation in female frequency in *C. palustre* and no significant effects in *C. heterophyllum* . Overall, our findings suggest that female persistence in the two studied *Cirsium* species partly reflects differences between polycarpic and monocarpic life‐history strategies, with reduced susceptibility to granivory providing modest reinforcement and environmental filtering playing a minimal role.

## Graphical abstract

We quantified how life history, reproductive traits, and environment shape female frequencies in two gynodioecious thistles, the polycarpic *Cirsium heterophyllum* and the monocarpic *C. palustre* , which differed strongly in female advantage. In both species, reproductive traits were the main drivers of female advantage, but their relative importance depended on life history: achene viability dominated in polycarpic *C. heterophyllum* , whereas increased floret production was most important in monocarpic *C. palustre* . Environmental variables contributed little, indicating that female persistence in *Cirsium* is governed primarily by life‐history–dependent reproductive advantages.

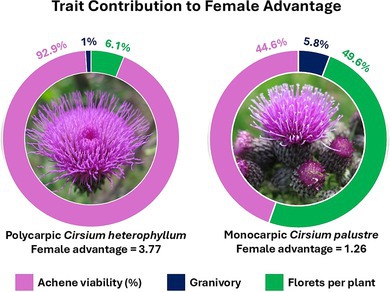

## 1 Introduction

Gynodioecy—the coexistence of female and hermaphroditic individuals within species—is a recurrent yet globally rare plant breeding system (< 1% of angiosperms; Godin and Demyanova 2013; Caruso et al. 2016; Rivkin et al. 2016; Godin 2020). The persistence of females in such systems is generally thought to require a reproductive advantage over hermaphrodites, often achieved through greater offspring quantity and/or quality. One commonly proposed mechanism involves the reallocation of resources saved through the loss of pollen production into increased seed production or other aspects of female function (Lewis 1941; Gavini 2023). However, female advantage may also arise through reduced inbreeding depression (Jolls and Chenier 1989; Dufay and Billard 2012), variation in resource availability and environmental stress (McCauley and Bailey 2009; Varga and Soulsbury 2020), or sex‐specific interactions with biotic and abiotic factors such as pollinator behavior and predispersal granivory (T.‐L. Ashman 2002; Asikainen and Mutikainen 2005; Collin et al. 2002; Stachurska‐Swakoń et al. 2018). The relative importance of these mechanisms may vary among gynodioecious systems depending on species life history and ecological context.

The maintenance of females in gynodioecious systems remains a central question in evolutionary biology, as females must compensate for the loss of male reproductive function through increased female fitness relative to hermaphrodites (Lloyd 1975; Jacobs and Wade 2003; McCauley and Bailey 2009; Dufay and Billard 2012). Classical theory predicts that females can persist when they achieve a sufficient fitness advantage through greater seed output, enhanced seed or offspring quality, or reduced reproductive losses mediated by ecological interactions such as seed predation (Lloyd 1975; Shykoff et al. 2003; Chang 2006; Dufay and Billard 2012; Clarke and Brody 2015; Varga 2021).

Ecological interactions can strongly modulate the balance of costs and benefits underlying the maintenance of gynodioecy (Asikainen and Mutikainen 2005). Such processes may enhance or erode female advantage by affecting the production of more and/or higher‐quality seeds, depending on how they interact with sex‐specific trait expression. Pollinator behavior may differentially affect male and female fitness (T.‐L. Ashman 2002; Asikainen and Mutikainen 2005), and predispersal granivory (seed predation) can remove a substantial fraction of developing seeds (Collin et al. 2002; Stachurska‐Swakoń et al. 2018). Importantly, if seed predators disproportionately target hermaphroditic plants, females may gain a relative fitness advantage despite lacking male function. Sex‐specific seed predation, particularly hermaphrodite‐biased granivory, has been documented across several unrelated gynodioecious taxa (Clarke and Brody 2015; Collin et al. 2002; Marshall and Ganders 2001; Miyake et al. 2018), suggesting that these ecological interactions may play an important role in maintaining gynodioecy. Although the potential importance of these interactions is widely recognized, explicit, system‐level tests linking life history, trait‐specific components of female advantage, and ecological context warrant further investigation.

Despite its rarity in Asteraceae (< 1%; Torices et al. 2011; Godin and Demyanova 2013; Godin 2020), gynodioecy is unusually frequent in the genus *Cirsium* Mill. Across Europe and East Asia, 71 species of *Cirsium* have been reported as gynodioecious (Demyanova 1985; Kawakubo 1995; Toshiaki 2006; Kadota 2007, 2008; Bureš et al. 2010, 2018; Kadota and Miura 2021; Michálková et al. 2023), representing ~14.4% of the genus species diversity (497 species: POWO 2025). Thistles were among the earliest systems in which male sterility and gynodioecy were documented (cf. Smith 1822; von Nägeli 1841; Juratzka 1857a, 1857b; Darwin 1877; MacLeod 1889; Knuth 1898; von Uexküll‐Gyllenband 1901; Correns 1916), yet have since received comparatively little attention in modern quantitative studies. The concentration of gynodioecy in *Cirsium*, contrasted with its overall scarcity in Asteraceae, together with the genus' predominance in temperate regions where gynodioecy is generally more frequent (Dufay and Billard 2012; Caruso et al. 2016), makes *Cirsium* a particularly suitable group for comparative analyses of female persistence. At the same time, female frequencies vary widely among *Cirsium* species and populations (Delannay 1978, 1979; Bureš et al. 2010), suggesting that the maintenance of females may depend on both genetic and ecological compensation mechanisms underlying female advantage. Theoretical models predict that females in systems with nuclear sex determination generally require approximately a two‐fold seed production advantage over hermaphrodites whereas substantially smaller advantages may be sufficient in cytonuclear systems (Charlesworth and Charlesworth 1978; Charlesworth 1981). Ecological mechanisms such as greater seed production, higher seed viability, and reduced losses to granivory may further enhance female fitness relative to hermaphrodites, with the relative importance of these mechanisms potentially differing among life‐history strategies and ecological contexts.

Predispersal granivory represents a key post‐pollination filter on reproductive success in many gynodioecious plant species, including *Cirsium* and related Carduoideae (van Leeuwen 1983; Klinkhamer et al. 1988; Zwölfer and Brandl 1989; Louda et al. 1990; Louda and Potvin 1995; Jump and Woodward 2003; Münzbergová 2005; Skuhrovec et al. 2008). The capitulum, often viewed as a key innovation contributing to the success of Asteraceae (Carlquist 1976; Mandel et al. 2019), also forms a predictable brood site for specialist granivores (e.g., tephritid flies, weevils, tortricid moths), which may consume many developing achenes (Zwölfer 1965; Redfern 1983; Fenner et al. 2002; Honek and Martinková 2005). Such losses can differ between females and hermaphrodites (e.g., *Cirsium oleraceum*; Čuprová 2014), depending on resource allocation, phenology, or capitulum traits, and may therefore contribute to sex‐specific differences in realized female advantage.

Environmental conditions further modulate the relative costs and benefits of female and hermaphroditic function by altering resource availability, plant performance, pollen limitation, or seed loss (Delph 2003; Delph and Wolf 2005; McCauley and Bailey 2009). Growing evidence suggests that environmental factors influence female frequency in gynodioecious systems, with higher female frequencies often associated with increased environmental stress, particularly temperature‐related factors (Varga and Soulsbury 2020). This pattern is consistent with the sex‐differential plasticity (SDP) hypothesis, which proposes that stressful or resource‐limited environments disproportionately reduce hermaphrodite reproductive performance, whereas females maintain reproductive output and consequently become more frequent under such conditions (Delph 2003; Van Etten and Chang 2009).

Climatic and edaphic gradients thus provide a natural setting in which to test how abiotic conditions and biotic pressures interact to influence female frequencies in gynodioecious species. Yet clear empirical tests remain limited, particularly in systems where female frequencies vary among populations within the same species (Dufay and Billard 2012).

Within *Cirsium*, truly extensive Eurasian distributions are rare (Bureš 2004; Del Guacchio et al. 2022; Bureš et al. 2023, 2024). *Cirsium heterophyllum* and *C. palustre* are two such species, sharing similar affinities for moist, cool habitats but differing markedly in traits linked to life history and resistance to herbivores. *Cirsium heterophyllum* is a polycarpic perennial with clonal growth, typically producing fewer but larger capitula on less robust shoots, and is nearly spineless (Bureš 2003). In contrast, *C. palustre* is monocarpic (biennial or short‐lived perennial), forming tall, vigorous shoots bearing many smaller capitula, and is among the most heavily armed species in the genus (Bureš 2004). These coordinated differences provide a natural contrast in life‐history and resource‐allocation strategies within a shared ecological framework.

While gynodioecy has been examined in ecological and genetic contexts, the role of life‐history strategies, particularly monocarpic versus polycarpic reproduction, in shaping female persistence remains largely unexplored. In polycarpic species, repeated reproductive episodes may allow females to accumulate advantages across years, whereas monocarpic species reproduce only once, potentially limiting opportunities for compensation (Herben et al. 2015; Klimešová et al. 2020). Differences in defense investment and capitulum architecture may further modify these dynamics by altering interactions with granivores and the distribution of resources between vegetative and reproductive structures. For instance, variation in capitulum size, number, or accessibility may influence susceptibility to predispersal seed predators (Fenner et al. 2002), whereas differences in defensive traits such as stem robustness and spines may also affect interactions with herbivores and resource allocation. This suggests that life history and associated traits could systematically influence the prevalence of females within populations, yet direct comparative evidence from closely related species is scarce.

To evaluate how life‐history strategy, trait‐specific female advantage, and environmental conditions jointly shape the maintenance of gynodioecy in *Cirsium*, we tested the following four hypotheses:

*Female frequencies are expected to differ between congeners with contrasting life histories, with the polycarpic Cirsium heterophyllum exhibiting higher female frequencies than the monocarpic C. palustre due to repeated reproductive allocation across multiple reproductive episodes*.

*Differences in female frequency between species and populations are expected to reflect differences in female advantage, with populations exhibiting higher female frequencies also showing stronger female advantage*.

*Trait‐specific components of female advantage, including vegetative performance, reproductive output, reproductive efficiency, and inbreeding‐related effects (achene production and viability), as well as reduced susceptibility to granivory, are expected to contribute differently to variation in female frequency within and between species*.

*Environmental conditions are expected to influence variation in female frequency across populations, with more stressful or resource‐limited environments associated with higher female frequencies*.

Together, these questions aim to clarify how life‐history strategy, reproductive and reduced susceptibility to granivory traits, and environmental context jointly interact to maintain female persistence in the studied gynodioecious *Cirsium*.

## 2 Material and Methods

### 2.1 Study Species

*Cirsium palustre* and *C. heterophyllum* are gynodioecious diploid species (2n = 34; Bureš et al. 2004) composed of female and hermaphroditic individuals. In both species, gynodioecy is recognized based on distinct floral morphology. Early crossing experiments by Correns (1916) indicated cytonuclear sex determination involving cytoplasmic male sterility (CMS) in *Cirsium*. However, the molecular basis of this system has not yet been confirmed in the genus.

*Cirsium palustre* is an insect‐pollinated, hemicryptophytic, monocarpic species lacking clonal growth and characterized by a multi‐year juvenile rosette phase. Mature rosettes produce a tall, spiny flowering shoot (up to 3 m) with hundreds of small capitula (50–150 florets each), after which the plant dies. The species is light‐demanding, occurs in moist, cool habitats, and ranges from the British Isles across Europe to western Siberia. *Cirsium heterophyllum* is an insect‐pollinated, hemicryptophytic polycarpic species with clonal growth via a horizontal to oblique rhizome. It forms sterile rosettes of soft, unarmed leaves, some developing into flowering shoots (60–125 cm) bearing 1–8 large capitula (140–390 florets). Flowering shoots die after reproduction, producing a mosaic of sterile and fertile ramets. The species also occupies moist, cool habitats and ranges from the British Isles across Europe to central Siberia and northern Mongolia (Meusel and Jäger 1992; Bureš 2004; Bureš et al. 2026).

**FIGURE 1:**
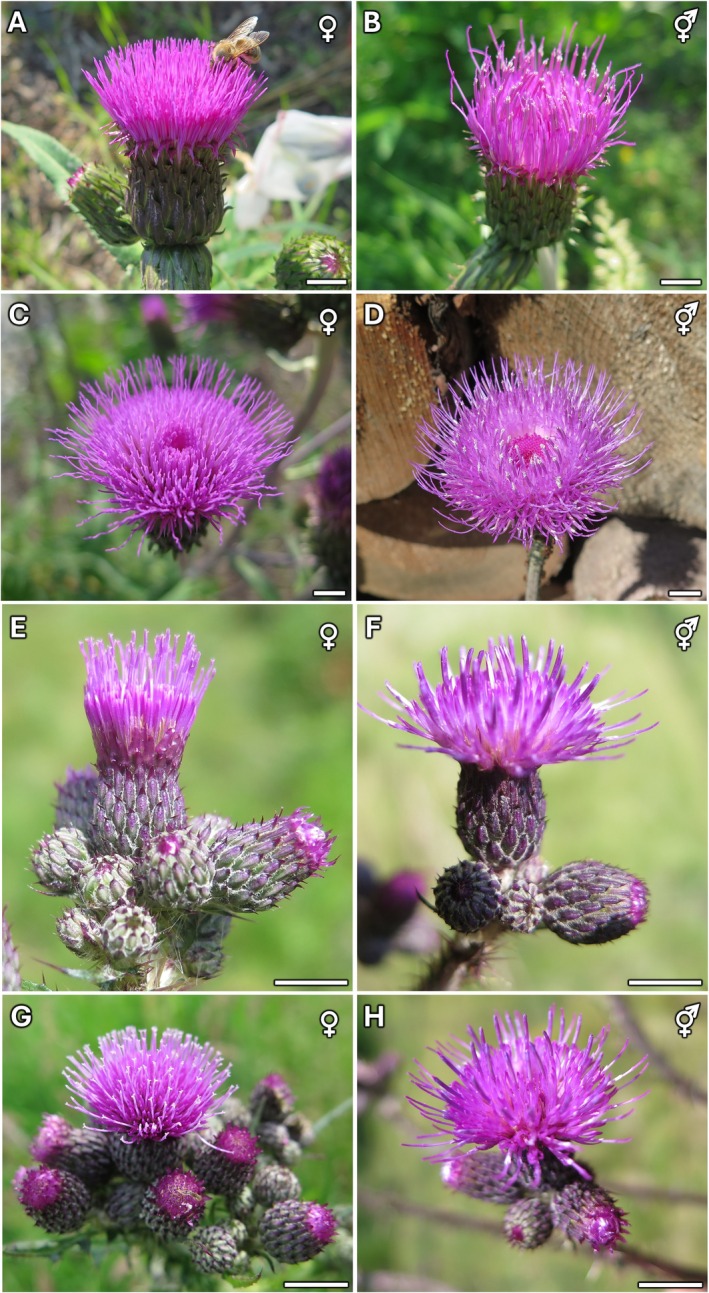
Female (A, C, E, G) and hermaphroditic (B, D, F, H) capitula of *Cirsium heterophyllum* (A–D) and *C. palustre* (E–H). Photo credits: Petr Bureš.

In both species, females and hermaphrodites differ clearly in floral morphology. Hermaphrodite florets produce an elongate anther tube that protrudes at anthesis and presents white pollen; stigmatic lobes remain appressed. Female (male‐sterile) florets have short, hidden anthers that never form a functional anther tube and produce no pollen; the style emerges freely and the stigmatic lobes are conspicuously spreading and wavy (Figure 1).

### 2.2 Sampling Strategy

To compare female frequencies between species and analyze their environmental correlates, we recorded hermaphrodites and females in 126 populations of *C. heterophyllum* (11,152 analyzed individuals) and 285 populations of *C. palustre* (16,398 analyzed individuals) across Austria, Czech Republic, Germany, France, Italy, Slovakia, Slovenia, and Switzerland from 2006 to 2025 (Supporting Information Set 1 and 2). The populations were sampled only once, and thus the dataset primarily represents spatial rather than temporal variation. In the clonal *C. heterophyllum* , individuals were recorded at ≥ 3 m spacing to minimize the likelihood of resampling the same genet.

For the detailed analysis of female advantage, vegetative and reproductive traits were sampled in 13 populations of *C. heterophyllum* (20 females and 20 hermaphrodites per population; 520 individuals; Supporting Information Set 3) and 16 populations of *C. palustre* (264 females and 264 hermaphrodites in total; Supporting Information Set 4). These populations were selected following the broader survey of female frequencies; consequently, sampling for female advantage was conducted subsequently and not necessarily in the same years as the female‐frequency surveys. In *C. palustre*, where females are rarer, sample sizes per population varied but always included equal numbers of females and hermaphrodites (10–20 each). The ≥ 3 m spacing rule was again applied to *C. heterophyllum* . All individuals were sampled at a standardized post‐flowering stage: when the terminal capitulum had closed after anthesis, shoot elongation had ceased, and achenes had formed but not yet matured. Flowering onset was not systematically recorded, but no consistent phenological differences between genders were observed during field sampling. The post‐flowering stage ensured comparable measurements of vegetative size, allowing for the subsequent assessment of achene traits and granivory following controlled maturation.

### 2.3 Recording and Measurement of Vegetative and Reproductive Traits

To analyze female advantage, we compared females and hermaphrodites for six traits measured at the standardized post‐flowering stage described above. Vegetative traits included (1) plant height, measured in the field, and (2) stem robustness, estimated as dry stem mass per cm. For stem robustness, 10‐cm stem segments collected from the basal and middle portions of the stem were dried for 96 h at 60°C and averaged per plant.

The remaining four traits—those related to florets, achenes, and granivores—were quantified after a 4‐week maturation period at 20°C in paper bags containing either (a1) the terminal capitulum and (a2) the lateral capitula of *C. heterophyllum* , or (b) a selected branch with capitula of *C. palustre* . After maturation, we recorded (3) the number of florets (developed + aborted achenes), (4) the number of developed achenes, and (5) the number of viable achenes (using tetrazolium chloride, TTC). Based on (4) and (5), the achene viability was calculated as the proportion (in %) of viable achenes relative to the total number of developed achenes. This metric was used to specifically assess post‐development achene viability independently of variation in floret fertilization and achene development. These per‐capitulum measurements were obtained (a) from the terminal capitulum of *C. heterophyllum* , or (b) as the mean of four fully developed, randomly selected capitula of *C. palustre* .

To estimate per‐plant values, these measurements were extrapolated as follows: (a) for *C. heterophyllum* , per‐capitulum values were multiplied by the ratio of the total dry mass of all capitula on the plant to the dry mass of the terminal capitulum; and (b) for *C. palustre* , per‐capitulum means were multiplied by the total number of capitula recorded in the field.

(6) Granivore abundance was assessed after the same maturation period, as the number of granivore individuals (adults and larvae) present in (a) the terminal capitulum of *C. heterophyllum* or (b) all capitula on the matured branch of *C. palustre* . Whole‐plant estimates were obtained using the same species‐specific extrapolation procedures: (a) dry‐mass ratios for *C. heterophyllum* and (b) multiplication by the ratio of the total number of capitula on the plant to the number of capitula on the sampled branch for *C. palustre* .

The full datasets, including locality, individual identity, sex, and all trait measurements, are provided in Supporting Information Sets 3 and 4.

### 2.4 Female Advantage Estimation

Female advantage was quantified as the ratio of the numbers of viable achenes per plant in females relative to hermaphrodites (ratios > 1 = female advantage). We evaluated female advantage (i) among populations within each species and (ii) between species.

### 2.5 Environmental Predictors of Female Frequency

For each population (126 populations of *C. heterophyllum* and 285 populations of *C. palustre* ), we extracted 22 environmental predictors: 19 bioclimatic variables (BIO1–BIO19) from the CHELSA and three soil variables from SoilGrids (pH in H_2_O, total nitrogen, and gravel volume at 0–5 cm depth).

To evaluate whether environmentally associated variation in female frequency was reflected in population‐level trait variation, we analyzed mean plant height, stem robustness, and granivory as proxy traits in *C. heterophyllum* and *C. palustre* (Tables S17 and S18).

### 2.6 Statistical Analyses

All statistical analyses were conducted in R (ver. 4.3.2; R Core Team 2023) within RStudio.

#### 2.6.1 Female Frequency and Female Advantage

Because female frequency and female advantage values deviated from normality, differences in female frequencies and female advantage between monocarpic *C. palustre* and polycarpic *C. heterophyllum* populations were tested using Wilcoxon rank‐sum tests. Boxplots were generated in R; in female frequency plots, point sizes were scaled to population sample sizes.

#### 2.6.2 Comparison of Female Advantage Traits Between Sexual Morphs

Sexual morph differences were evaluated using linear mixed‐effects models (LMMs) for normally distributed continuous traits and generalized linear mixed‐effects models (GLMMs) for non‐normal or discrete traits, implemented with lme4 (lmer/glmer; Bates et al. 2015; Kuznetsova et al. 2017).

According to the two‐way ANOVA, plant height differed significantly among populations (*p* < 0.001), although significant sex differences were detected in only two populations of *C. heterophyllum* and in none of the *C. palustre* populations based on Wilcoxon rank‐sum tests (Figures S3 and S4). The pronounced among‐population differences highlight the importance of a balanced sampling design—with equal numbers of females and hermaphrodites per population—to reliably distinguish population effects from sex effects and accurately assess sex‐specific trait variation. In the subsequent analysis, sex was included as a fixed effect and population (locality) as a random effect to account for the among‐population variation.

Plant height and other continuous traits (e.g., stem robustness) were analyzed using LMMs. Count traits (e.g., florets per plant, number of viable achenes per plant) were fitted using Poisson GLMMs. The achene viability (proportion of viable achenes in developed achenes) was analyzed using a binomial GLMM with a two‐column response (cbind(viable achenes, developed achenes − viable achenes)), followed by back‐transformation to percentages. Model adequacy was checked by visual inspection of residuals.

#### 2.6.3 Effect of Stem Robustness on Florets Per Plant

To test whether reproductive investment scales with plant structural investment, we analyzed the relationship between stem robustness and florets per plant in both *C. heterophyllum* and *C. palustre* . Stem robustness was standardized (z‐transformed) and included as a predictor in Poisson GLMMs, together with sex and their interaction (Sex × Robustness), while locality was included as a random intercept. For both species, the interaction model provided a significantly better fit than the additive model (Tables S13 and S15). Because florets per plant scaled strongly with stem robustness (Figure S2; Tables S14 and S16), robustness was not included as a separate predictor in subsequent analyses of female advantage. Instead, florets per plant were used as the primary measure of reproductive investment.

#### 2.6.4 Trait Contribution to Female Advantage

To identify which trait components contribute to species‐level differences in female advantage, we fitted linear models separately for *C. heterophyllum* and *C. palustre* . Female advantage, calculated for each population as described in Section 2.4, was used as the response variable. Population‐level trait ratios were included as predictors (Tables S9 and S10): (1) florets per plant (female: hermaphrodite ratio; a proxy for resource allocation to reproductive output), (2) achene viability (female: hermaphrodite ratio; used as a proxy for post‐development reproductive performance, potentially reflecting processes associated with reproductive fitness, including inbreeding‐related effects), and (3) granivory disadvantage (hermaphrodite: female ratio; used as a proxy for differential susceptibility to granivory between sexual morphs, potentially reflecting differences in defense related traits).

To quantify the unique contribution of each trait to variation in female advantage, we applied a Δ*R* ^2^ (delta‐*R* ^2^) partitioning approach. For each species, the *R* ^2^ of the full model was compared with that of reduced models in which one predictor at a time was omitted. Because female advantage is influenced by multiple reproductive and ecological trait components, the predictors analyzed here should be interpreted as partially related components contributing to female advantage rather than as fully independent predictors. The resulting Δ*R* ^2^ values represent the relative contribution of each trait component to variation in female advantage explained by each trait. These values were rescaled to sum to 100% for graphical presentation.

#### 2.6.5 Environmental Predictors

Relationships between female frequency and environmental variables were evaluated using single‐predictor weighted least‐squares (WLS) regressions for each species, with log‐transformed population sample size as a weighting factor to account for differences in precision.

To address the correlation among the 22 environmental variables, we estimated the effective number of independent predictors (Meff = 7.07; Li and Ji 2005) and identified seven independent predictor clusters based on their correlation structure, visualized using a Pearson correlation heat map (Figure S7). A Sidák‐corrected significance threshold of *p* < 0.007 (familywise α = 0.05) was applied to reduce false positives while retaining power to detect genuine associations.

Relationships between environmental variables and population‐level trait means (plant height, stem robustness, and granivory) were evaluated using linear regressions in *C. heterophyllum* and *C. palustre* .

## 3 Results

### 3.1 Female Frequencies

**FIGURE 2:**
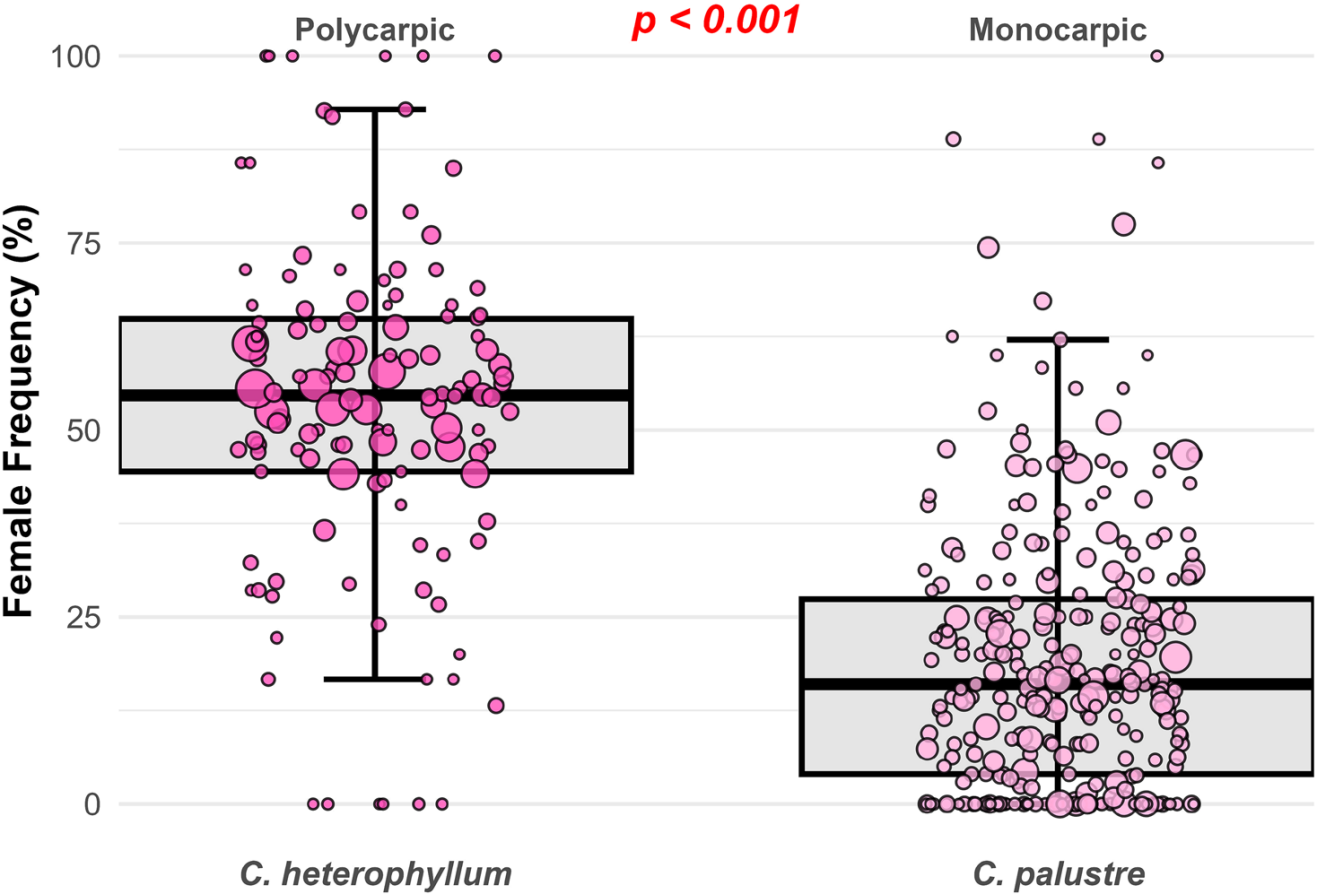
Female frequency in populations of *Cirsium heterophyllum* and *C. palustre* . Boxplots represent the distribution of female frequency across 126 and 285 populations for each species, respectively. Point size is proportional to the number of individuals sampled per population (sampling effort) and should not be interpreted as an estimate of census population size. Boxes represent the interquartile range, with the median indicated by the thick horizontal line ( *C. heterophyllum* : Median = 54.6%; *C. palustre* : Median = 16.0%). Significant sex differences (Wilcoxon rank‐sum test; *p* < 0.001) are shown in red.

Female frequencies differed significantly between the polycarpic *C. heterophyllum* and the monocarpic *C. palustre* (Wilcoxon rank‐sum test, *p* < 0.001; Figure 2). Female frequency was higher in *C. heterophyllum* (mean = 53.3%; median = 54.6%; 126 populations; 11,152 individuals) than in *C. palustre* (mean = 19.3%; median = 16.0%; 285 populations; 16,398 individuals).

### 3.2 Female Advantage

**FIGURE 3:**
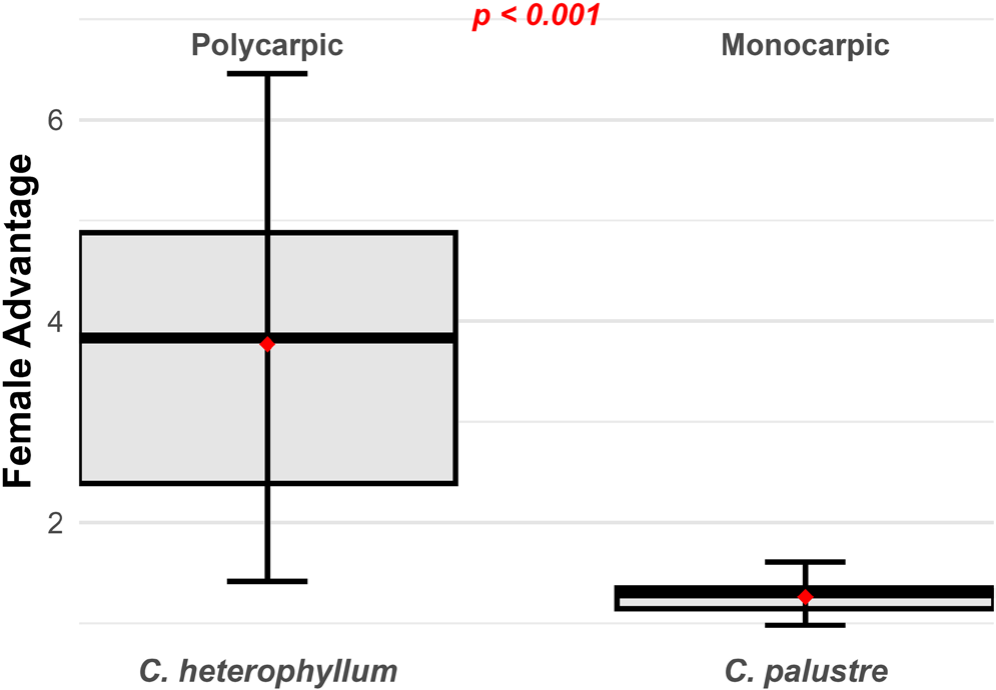
Female advantage in *Cirsium heterophyllum* and *C. palustre* —population level. Boxplots show the distribution of female advantage (viable achenes per plant in females relative to hermaphrodites) across populations for each species. Boxes represent the interquartile range, with the median indicated by the thick horizontal line ( *C. heterophyllum* : Median = 3.83; *C. palustre* : Median = 1.30) and red diamonds indicate means ( *C. heterophyllum* : Mean = 3.77; *C. palustre* : Mean = 1.26). Significant sex differences (Wilcoxon rank‐sum test; *p* < 0.001) are shown in red.

**FIGURE 4:**
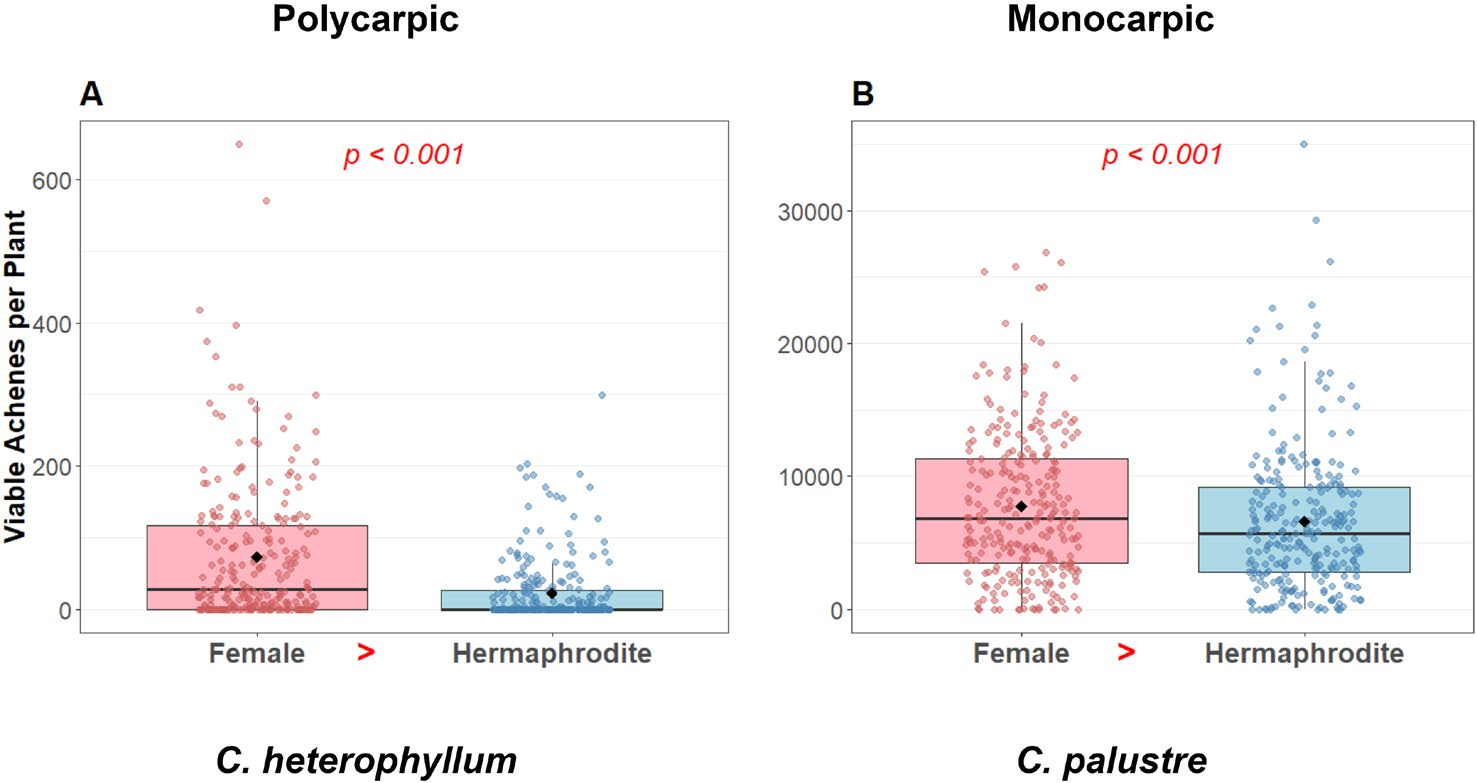
Female advantage in *Cirsium heterophyllum* and *C. palustre* —individual level. Comparison of viable achenes per plant between female and hermaphroditic individuals of *C. heterophyllum* (A) and *C. palustre* (B). Significant sex differences based on generalized linear mixed‐effects model (GLMM; *p* < 0.05) are shown in red. When differences are significant, the larger sex is denoted with “>” between females (F) and hermaphrodites (H). Observed viable achenes per plant means (± SE) were as follows: *C. heterophyllum* —viable achenes: F = 72.40 ± 6.14, H = 22.40 ± 2.78. *C. palustre* —viable achenes: F = 7768 ± 344, H = 6622 ± 339.

Female advantage was quantified (i) at the population level as the ratio of the numbers of viable achenes per plant in females relative to hermaphrodites, and (ii) at the individual level as sex differences in viable achenes per plant; both approaches yielded consistent results. At the population level, female advantage showed a significant difference between the two species (Wilcoxon rank‐sum test, *p* < 0.001; Figure 3), being high in all *C. heterophyllum* populations, whereas in *C. palustre* it was generally only slightly above 1 except in two populations (Tables S1 and S2). At the species level, females of *C. heterophyllum* showed a greater female advantage (3.77) than those of *C. palustre* (1.26). At the individual level, viable achenes per plant were higher in females compared to hermaphrodites in both *C. heterophyllum* and *C. palustre* (*p* < 0.001; Figure 4; Table S6). However, linear regression analyses revealed no significant population‐level association between female frequency and female advantage in either *C. heterophyllum* (adjusted *R* ^2^ = −0.017, *p* = 0.391) or *C. palustre* (adjusted *R* ^2^ = −0.026, *p* = 0.443).

### 3.3 Female Advantage in Particular Traits

To identify the traits contributing to female advantage, we quantified sex differences across five functional components: vegetative performance, reproductive output, reproductive efficiency, inbreeding‐related effects, and reduced susceptibility to granivory (defense). Inbreeding‐related effects were not measured directly through controlled crossing experiments but were inferred indirectly using sex differences in achene viability as a proxy.

**FIGURE 5:**
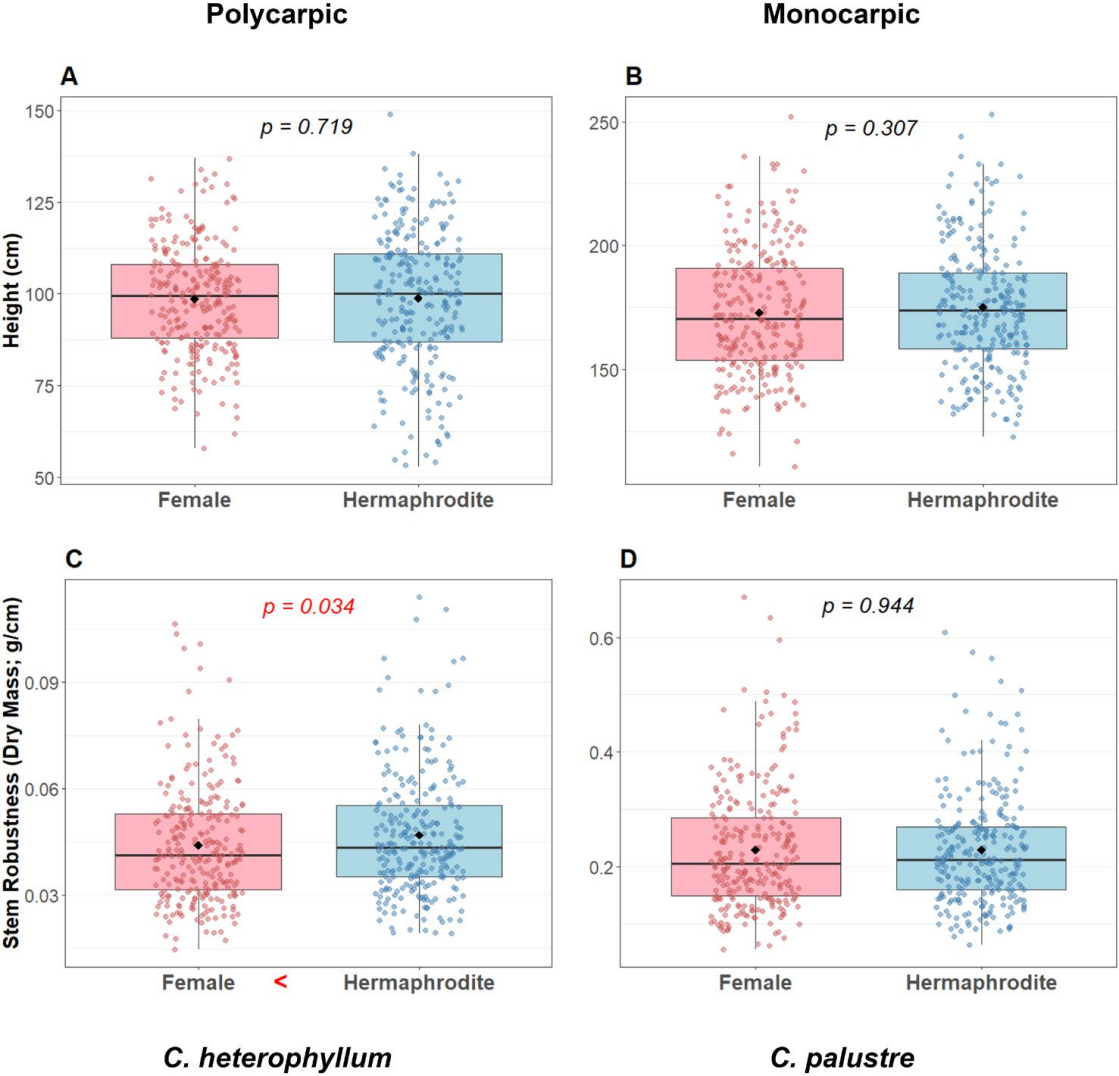
Comparison of vegetative traits (related to resource allocation to plant performance) between female and hermaphroditic individuals of polycarpic *Cirsium heterophyllum* (A, C) and monocarpic *C. palustre* (B, D). Plant height (in cm; A, B). Stem robustness (dry mass, g cm^−1^; C, D). Boxes show the interquartile range with the median represented by the thick black horizontal line; points represent individual plants, and black diamonds indicate means. Significant sex differences based on linear mixed‐effects models (LMMs; *p* < 0.05) are shown in red. When differences are significant, the larger sex is denoted with “<” between females (F) and hermaphrodites (H). Observed height and stem‐robustness means (± SE) for F and H were as follows: *C. heterophyllum* —height: F = 98.60 ± 0.92 cm, H = 98.80 ± 1.14 cm; robustness: F = 0.044 ± 0.001 g cm^−1^, H = 0.047 ± 0.001 g cm^−1^. *C. palustre* —height: F = 173.00 ± 1.55 cm, H = 175.00 ± 1.51 cm; robustness: F = 0.229 ± 0.007 g cm^−1^, H = 0.229 ± 0.006 g cm^−1^.

Vegetative performance showed little evidence of sex‐specific differentiation and therefore appeared to contribute minimally to female advantage in either species. Plant height did not differ significantly between females and hermaphrodites in either *C. heterophyllum* or *C. palustre* (*p* > 0.05; Figure 5A,B; Table S3). Similarly, no sex differences in stem robustness were detected in *C. palustre* (Figure 5D). In *C. heterophyllum* , however, hermaphrodites had slightly stronger stems (0.047 ± 0.001 g/cm) than females (0.044 ± 0.001 g/cm; *p* = 0.034; Figure 5C; Table S4).

**FIGURE 6:**
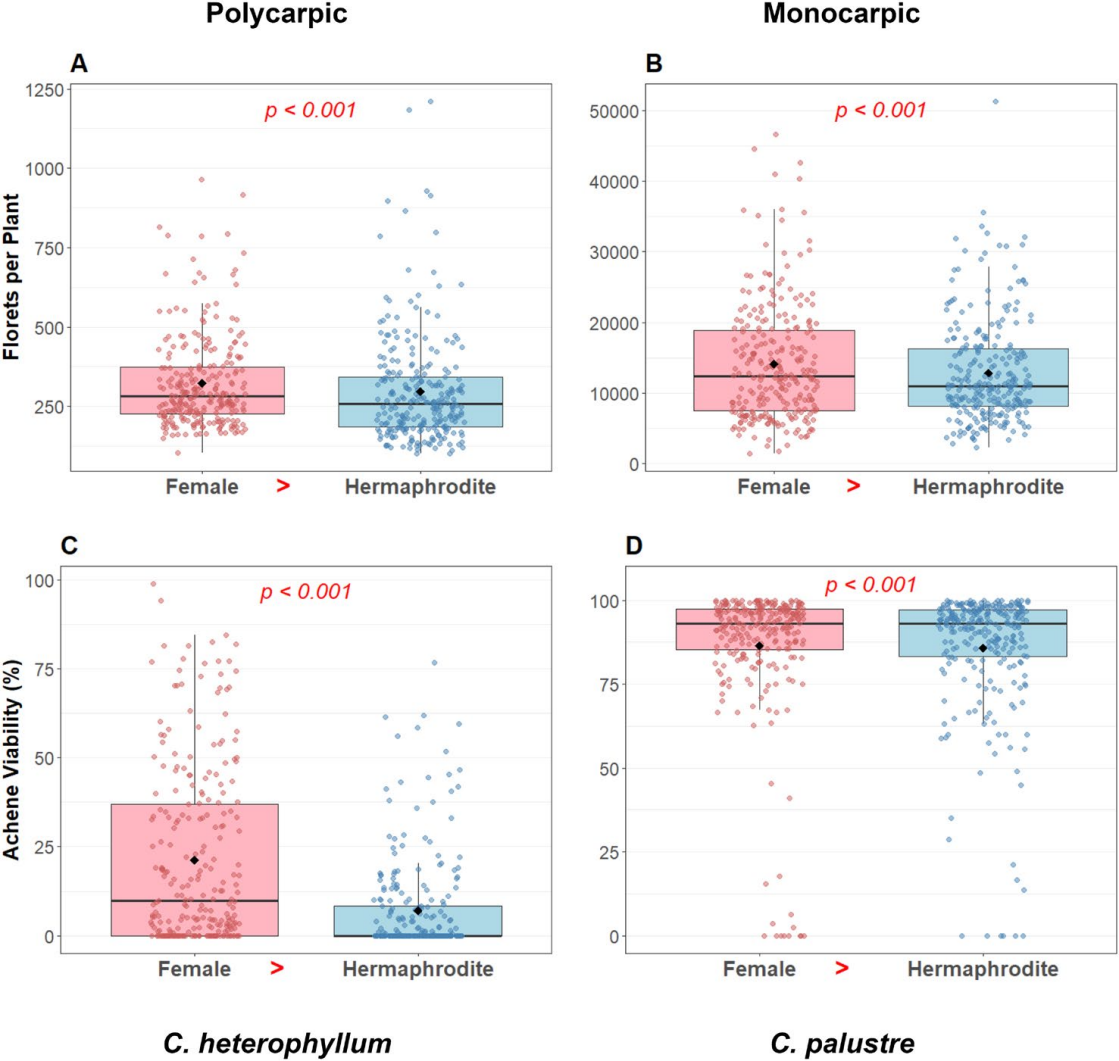
Comparison of reproductive traits between female and hermaphroditic individuals of polycarpic *Cirsium heterophyllum* (A, C) and monocarpic *C. palustre* (B, D). Number of florets per plant (A, B). Percentage of achene viability (C, D). Significant sex differences on generalized linear mixed‐effects models (GLMMs; *p* < 0.05) are shown in red. When differences are significant, the larger sex is denoted with “>” between females (F) and hermaphrodites (H). Observed florets and viable achenes per plant means (± SE) for females (F) and hermaphrodites (H) were as follows: *C. heterophyllum* —florets: F = 323 ± 9.04, H = 296 ± 10.4; achene viability: F = 21.30 ± 1.55, H = 6.99 ± 0.83. *C. palustre* —florets: F = 14,100 ± 510, H = 12,850 ± 444; achene viability: F = 86.5 ± 1.31, H = 85.7 ± 1.22.

In contrast, reproductive traits consistently favored females in both species. Females produced significantly more florets per plant than hermaphrodites (*p* < 0.001; Figure 6A,B; Table S5) and also exhibited a higher proportion of viable achenes (*p* < 0.001; Figure 6C,D; Table S7).

**FIGURE 7:**
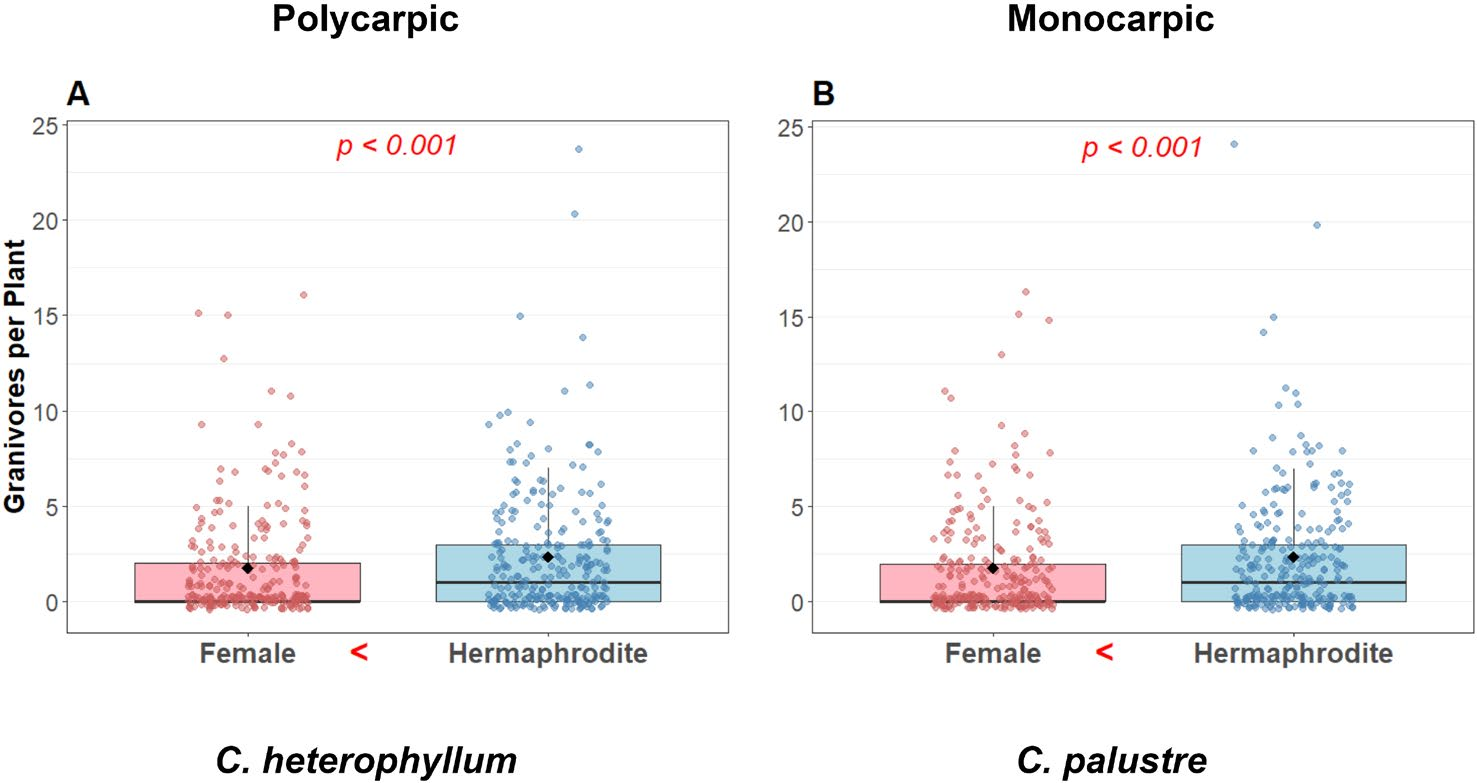
Comparison of granivore (weevils and tephritid flies) numbers per plant between female and hermaphroditic individuals in polycarpic *Cirsium heterophyllum* (A) and monocarpic *C. palustre* (B). Significant sex differences on generalized linear mixed‐effects models (GLMMs; *p* < 0.05) are shown in red, with the larger sex indicated by “<” between females (F) and hermaphrodites (H). Observed granivores per plant means (± SE) were as follows: *C. heterophyllum* —F = 1.75 ± 0.17, H = 2.33 ± 0.20; *C. palustre* —F = 1.72 ± 0.19, H = 2.30 ± 0.20.

**FIGURE 8:**
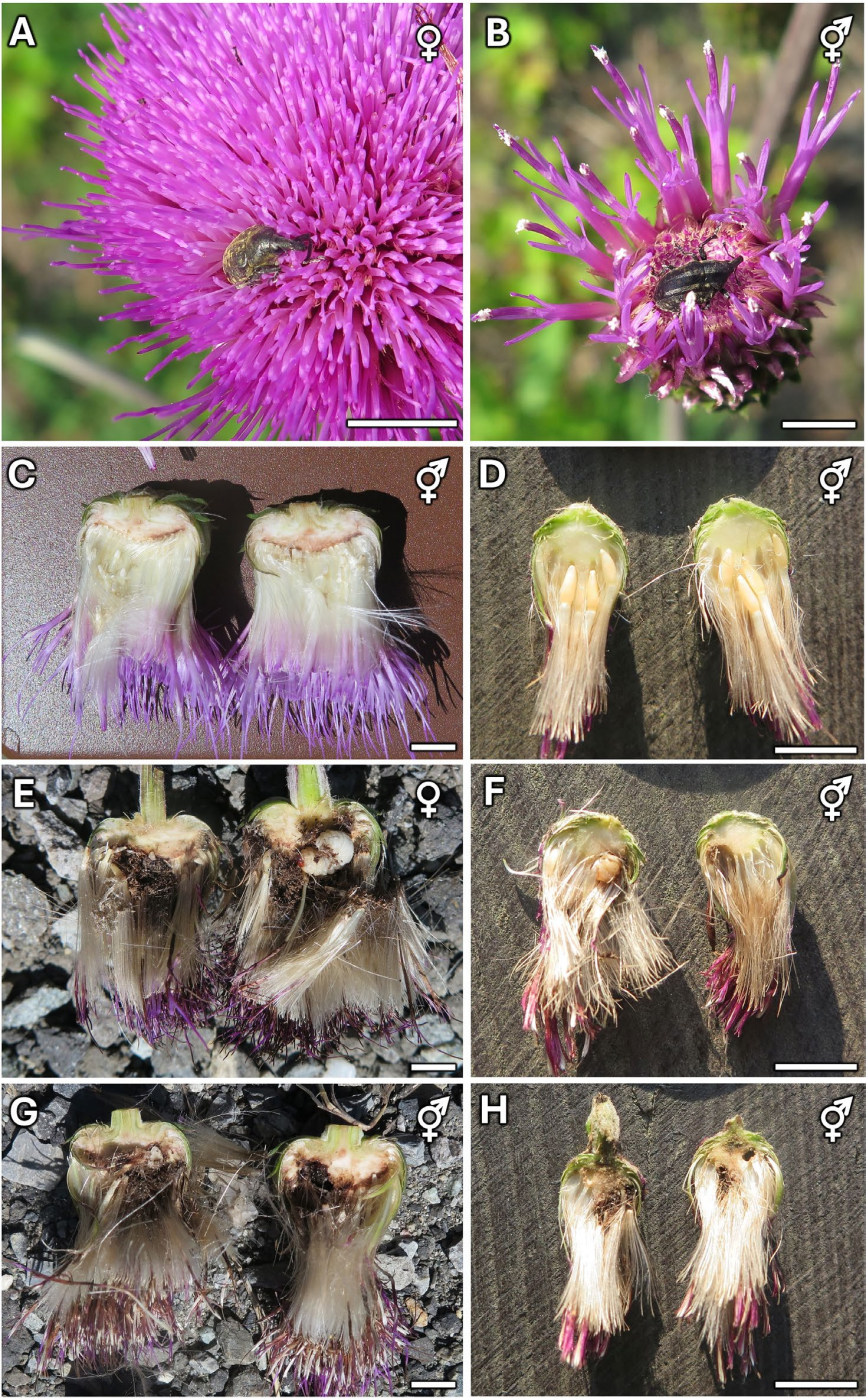
Pre‐dispersal granivory in polycarpic *Cirsium heterophyllum* (A, C, E, G) and monocarpic *C. palustre* (B, D, F, H). A, B, Adult weevil on capitula; C, D, healthy, uninfested capitula; E–H, capitula infected by weevil larvae showing internal damage to achenes and receptacle tissue. ♀ = female capitulum; ⚥ = hermaphrodite capitulum. Species‐level identifications of the granivores are provided in Figure S8. Photo credits: Petr Bureš.

Patterns of predispersal achene loss also differed between sexual morphs. Hermaphrodites harbored significantly more granivores per plant than females in both species (*p* < 0.001; Figure 7A,B; Table S8). Granivores (Figure S8) utilized *Cirsium* capitula as brood sites, and larval feeding damaged both achenes and receptacle tissue (Figure 8).

### 3.4 Trait Contributions to Female Advantage

**FIGURE 9:**
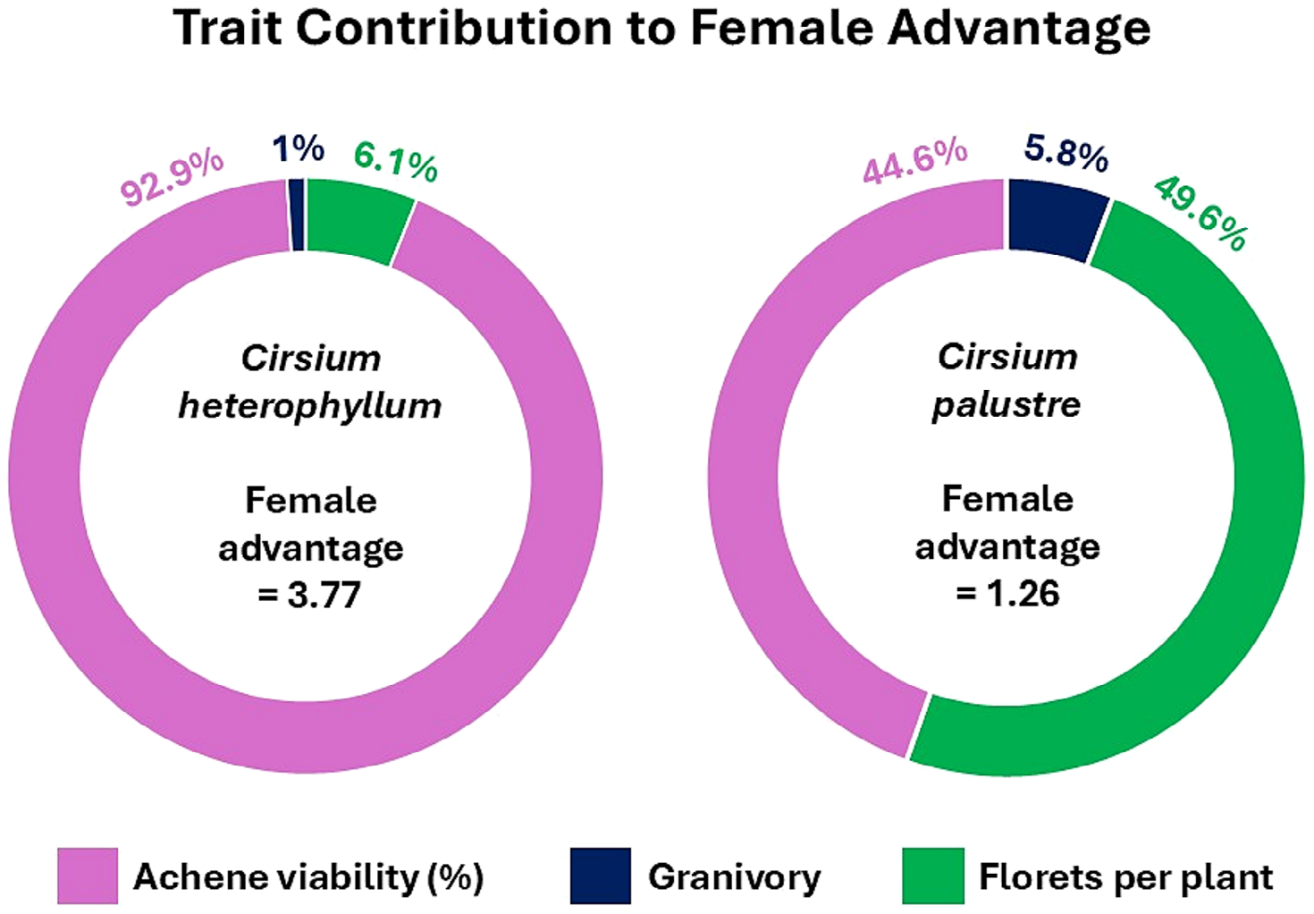
Trait contributions to female advantage in polycarpic *Cirsium heterophyllum* (left) and monocarpic *C. palustre* (right). Doughnut charts show the unique contributions (Δ*R* ^2^) of three sex‐specific trait ratios to female advantage: (i) floret number ratio (F/H; resource allocation), (ii) achene viability ratio (F/H; reproductive fitness and inbreeding depression), and (iii) granivory disadvantage ratio (H/F; defense). Each segment represents the change in explained variance obtained by removing the focal trait from the full regression model. These proportions correspond to the unique variance values in Table S11. In *C. heterophyllum* , the combination of three trait ratios (full model) explains 0.801 (80.1%) of the variation in female advantage, of which 0.554 (55.4%) reflects their unique contributions (sum of Δ*R* ^2^). In *C. palustre* , the combination of these three traits explains 0.255 (25.5%) of the variation, with 0.15 (15%) attributable to their unique contributions.

Trait contributions to female advantage were quantified as the reduction in explained variance (Δ*R* ^2^) resulting from removal of each focal predictor from the full regression model. The magnitude of traits contributing to female advantage differed markedly between the polycarpic *C. heterophyllum* and the monocarpic *C. palustre* (Figure 9). In *C. heterophyllum* , achene viability accounted for nearly all proportional contributions of relative explained variance in female advantage (Δ*R* ^2^ = 92.9%), whereas florets per plant (Δ*R* ^2^ = 6.1%) and granivory (Δ*R* ^2^ = 1%) played only minor roles. In contrast, in *C. palustre* , florets per plant explained most variance (Δ*R* ^2^ = 49.6%), followed by achene viability (Δ*R* ^2^ = 44.6%) and granivory (Δ*R* ^2^ = 5.8%).

### 3.5 Environmental Predictors of Female Frequency

Among the 22 environmental predictors, BIO4 (Temperature seasonality) was the only variable showing a statistically significant relationship with female frequency in *Cirsium palustre* , explaining 6.5% of the variation (*β* = 0.0225, *R* ^*2*^ = 0.065, raw *p* < 0.001, adjusted *p* < 0.001; see Table S22).

In contrast, none of the 22 environmental variables were significantly associated with female frequency in *C. heterophyllum*. All models exhibited low explanatory power (*R* ^2^ ≈ 0.01–0.03) and adjusted *p*‐values > 0.05, indicating that environmental variation did not significantly explain the observed differences in female frequency among populations (Table S21).

## 4 Discussion

Our results suggest that contrasting life‐history strategies (monocarpic vs. polycarpic) may partially contribute to the observed differences in female frequencies between the two studied gynodioecious *Cirsium* species. Previous studies on female persistence in gynodioecious species have primarily examined other dimensions of variation, including cytotype differences in perennial clonal species (e.g., diploid vs. tetraploid *Stellaria graminea* ; Kučera et al. 2021) and variation in life‐cycle strategies (annual, biennial, and perennial species; Dufay and Billard 2012). To our knowledge, this study is among the first to investigate female advantage in relation to contrasting monocarpic and polycarpic life‐history strategies in two gynodioecious *Cirsium* species. Female frequencies differed strongly between the polycarpic *C. heterophyllum* and the monocarpic *C. palustre* , corresponding to species‐level differences in female advantage observed in this study. Trait‐level analyses further revealed sexual‐morph differentiation in both reproductive and granivory traits across the two species. However, these findings are limited to the species examined here, and further comparative studies across additional *Cirsium* and other gynodioecious species are needed to evaluate whether similar patterns occur more broadly.

Across the large geographic dataset comprising 285 populations of *C. palustre* and 126 populations of *C. heterophyllum* , female frequencies differed not only in their mean values but also in their population‐level distributions. Although both species occasionally included entirely female and entirely hermaphroditic populations, *C. palustre* showed substantially greater heterogeneity among populations, including many populations with very low female frequencies or lacking females entirely. In contrast, female frequencies in *C. heterophyllum* were more consistently centered around intermediate to high values. These patterns suggest that the maintenance of gynodioecy may be more stable in the studied polycarpic *C. heterophyllum* than in the monocarpic *C. palustre* .

Species‐level means of female advantage can match theoretical expectations because they mask substantial population‐level variation. The absence of a population‐level association between female advantage and female frequency in our system likely reflects several processes. High female frequencies may arise independently of balancing selection; for example, repeated introductions of novel cytoplasmic male sterility (CMS) types can inflate female frequencies in *Lobelia siphilitica* (Campanulaceae) (Adhikari et al. 2019), and CMS introgression via hybridization—as indicated by Correns' early *Cirsium* experiments (1916)—could similarly elevate female frequencies without current fitness advantages. Because CMS is maternally inherited, backcrossing restores the nuclear genome of the pollen donor but retains the sterility factor, producing morphologically typical yet male‐sterile plants; such cytoplasmic introgression, whether interspecific or among populations, can raise female frequencies irrespective of present‐day advantage. In cytonuclear gynodioecious systems, variation in the cost of male fertility restoration may also contribute to variation in female frequency (Caruso and Case 2013), potentially weakening the expected relationship between female advantage and female frequency. Although pollen viability may provide an indirect proxy for the cost of male fertility restoration, confirming whether restoration costs or CMS dynamics contribute to sex‐ratio variation in *Cirsium* will require future genetic and experimental studies.

In addition, theoretical models of gynodioecy predict that the relationship between female advantage and female frequency may be frequency dependent rather than strictly positive. At high female frequencies, pollen limitation may generate negative frequency‐dependent selection, resulting in temporal fluctuations in sex ratios and weakening simple population‐level associations between female advantage and female frequency (Dufay and Billard 2012). Because pollination success was not quantified in this study, we were unable to evaluate the importance of these processes directly. These dynamics may help explain the absence of a significant population‐level relationship between female advantage and female frequency within either species, despite the broadly consistent species‐level differences observed between *C. heterophyllum* and *C. palustre* . In *C. heterophyllum* , repeated sampling of ramets belonging to the same genet cannot be fully excluded without molecular identification. However, sampling was conducted across spatially separated patches, and individuals were sampled at ≥ 3 m spacing to reduce the likelihood of repeatedly sampling the same clone. While some degree of clonity‐related non‐independence may still occur, especially in extensive clonal systems, and may have influenced female‐frequency estimates in some populations, the broad geographic and population‐level consistency of observed patterns suggests that higher female frequencies in *C. heterophyllum* are unlikely to be explained solely by clone resampling.

Alternatively, demographic buffering may decouple sex ratios from current selection: clonality in *C. heterophyllum* (Bureš et al. 2026) and seed bank dynamics in *C. palustre* (Falińska 1997) can slow population‐level responses to female advantage. Although environmental predictors explained little variation in female frequency in our analyses, particularly in *C. heterophyllum* , with only weak support detected for temperature seasonality in *C. palustre* , resource availability may nevertheless mask sex‐differential performance (a ceiling effect), with both sexes performing near optimally in resource‐rich habitats and female advantage becoming more pronounced only under resource limitation (Delph 2003; Putney et al. 2022). Taken together, these processes offer plausible explanations for why female advantage and observed female frequencies are decoupled at the population level.

### 4.1 Female Frequencies and Life History

Female frequency was higher in polycarpic *C. heterophyllum* than in monocarpic *C. palustre* . While gynodioecy can occur in monocarpic *Cirsium* species, including *C. palustre* and *C. brachycephalum* (Bureš et al. 2004, 2010), it appears to be more widespread among polycarpic *Cirsium* species. Previous work has shown that reproductive allocation and sexual dimorphism vary according to life‐history strategy, including differences between monocarpic and short‐lived polycarpic species (Lloyd and Webb 1977; Obeso 2002; Barrett and Hough 2013). This pattern suggests that life‐history strategy may influence the maintenance of gynodioecy within the *Cirsium* taxa examined here. Because monocarpic taxa channel most resources into a single terminal reproductive event (Amasino 2009), the persistence of female morphs may depend on sufficiently large female fitness advantages being realized during that reproductive episode. Consistent with this life‐history strategy, *C. palustre* produced more florets per plant than the polycarpic *C. heterophyllum* , indicating greater reproductive investment in a single flowering event. We therefore hypothesize that the maintenance of gynodioecy may be more difficult in monocarpic than in polycarpic *Cirsium*, although broader comparative studies across additional taxa are needed to test this hypothesis.

### 4.2 Female Advantage and Trait Contributions

**FIGURE 10:**
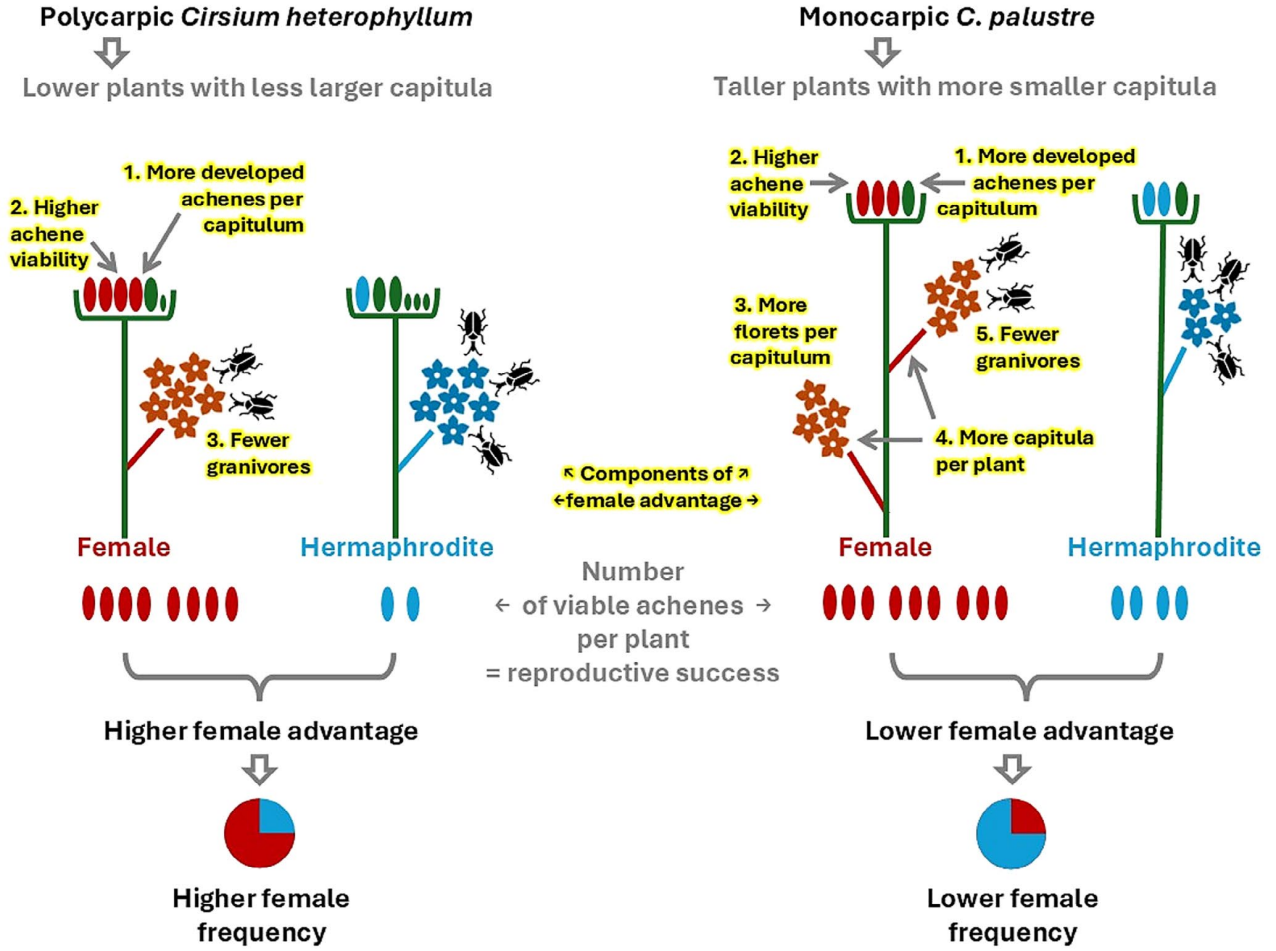
Summary of life‐history traits, plant performance, and components of female advantage in gynodioecious *Cirsium heterophyllum* (left) and *C. palustre* (right). *C. heterophyllum* (polycarpic) forms shorter plants with fewer but larger capitula; females show higher achene viability, more developed achenes per plant, and reduced granivory, resulting in a strong female advantage and higher female frequency. In contrast, *C. palustre* , a monocarpic species, produces taller plants with more but smaller capitula, and females with more florets per capitulum (Figure S1; Table S12), higher achene viability, more developed achenes, and reduced granivory; however, the overall female advantage is weaker, leading to a lower female frequency.

Female advantage in both species arose chiefly from reproductive traits, reinforcing the idea that vegetative dimorphism plays only a minor role in gynodioecious systems (Obeso 2002; T.‐L. Ashman 2005). Vegetative trait differences were minimal, aside from slightly thicker stems in hermaphrodites of *C. heterophyllum* , perhaps reflecting the structural demands of supporting both pollen and achene production. Larger floral displays in hermaphrodites of *C. heterophyllum* resemble patterns in other gynodioecious taxa, where male‐function investment is often associated with enhanced pollinator attraction (Eckhart 1999; Shykoff et al. 2003). The greater granivore abundance observed in hermaphrodites may likewise be linked to increased attractiveness or accessibility of reproductive structures, although this pattern is unlikely to be explained solely by larger capitula because females produced more florets per plant in both species. In contrast to *C. heterophyllum* , floral display size did not differ between female and hermaphrodite morphs in *C. palustre* . This may indicate that high female floret production necessitates robust stems, thereby reducing opportunities for sex‐specific vegetative divergence. Alternatively, differences in resource‐allocation timing may be key: monocarpic *C. palustre* invest heavily only after floral primordia initiate, as our results show that females of the monocarpic *C. palustre* produced significantly more capitula per plant than hermaphrodites (Figure S1; Table S12), whereas no such difference was detected in the polycarpic *C. heterophyllum* . Therefore, in *C. palustre* , the higher total number of florets per plant in females reflects the combined effect of producing more capitula and more florets per capitulum (Figure 10). Although developed for dioecious systems, theoretical work suggests that pollinator‐mediated selection and pollen limitation can influence the evolution of floral sexual dimorphism (Vamosi and Otto 2002). Whether similar mechanisms contribute to floral trait divergence between females and hermaphrodites in gynodioecious taxa remains to be investigated.

Reproductive traits consistently favored females, but their relative contributions differed with life history. Because achene viability was used as an indirect proxy for inbreeding‐related effects, these results suggest reduced inbreeding‐related fitness costs in females relative to hermaphrodites. In polycarpic *C. heterophyllum* , elevated achene viability was the main driver of female advantage, consistent with the higher offspring quality and/or quantity expected from obligate outcrossing and reduced inbreeding depression (Darwin 1877; Lewis 1941; Emery and McCauley 2002; Dufay and Billard 2012; Stone and Olson 2018). This benefit may be diminished when outcrossing occurs among close relatives, resulting in biparental inbreeding (Chang 2007). In monocarpic *C. palustre* , by contrast, increased floret production contributed most strongly to female advantage, reflecting the need to maximize reproductive output within a single flowering episode.

Our results show that lower predispersal granivory also contributed to female advantage, though only modestly (5.8% in *C. palustre* and 1% in *C. heterophyllum* ). Hermaphrodites suffered greater damage from achene‐feeding insects, consistent with patterns in other gynodioecious species (Marshall 1988; Asikainen and Mutikainen 2005; Caruso and Case 2007; Collin et al. 2002; Clarke and Brody 2015; Miyake et al. 2018). Such preferences are often mediated by floral traits—including morphology, scent, nectar rewards, and other chemical cues—that influence attraction and susceptibility to predation (El‐Sayed et al. 2008; T. L. Ashman 2009; Schiestl 2010), although their sex specificity requires further verification. Several hermaphroditic *Cirsium* species have previously been reported as important nectar sources for butterflies and other insects, including *C. vulgare* , *C. altissimum* , *C. discolor* , *C. pumilum* (Robertson 1928), and *C. wallichii* (Boruah et al. 2020). However, sex‐specific differences in nectar production or nectar gland morphology have not yet been studied in detail in Central European gynodioecious *Cirsium* species. Sticky glandular secretions of involucral bracts may additionally contribute to defense against ovipositing insects in *Cirsium*. Because granivore abundance was assessed at a standardized post‐flowering stage rather than repeatedly across the flowering season, temporal variation in infestation among capitula positions or flowering cohorts may not have been fully captured.

### 4.3 Female Frequency and Environmental Predictors

Neither female advantage nor female frequency was consistently associated with environmental predictors among populations within *C. heterophyllum* or *C. palustre* (Tables S19 and S20). Environmental proxies, including plant height, stem robustness, and granivory, measured at the population level, showed no consistent relationships with either variable. Although stem robustness significantly affected female advantage in *C. palustre* and plant height marginally influenced female frequency in *C. heterophyllum* , these isolated effects did not reflect broader patterns. Similarly, population‐level comparisons revealed no association between female advantage and female frequency (Tables S1 and S2).

Climatic and edaphic variables explained only a small proportion of the variation in female frequency. Temperature seasonality (BIO4) had a modest but significant effect in *C. palustre* (6.5%), whereas no environmental predictors were significant in *C. heterophyllum* . Importantly, these weak effects are unlikely to reflect limited environmental coverage, as both species were sampled across broad and nearly continuous elevational gradients ( *C. heterophyllum* 126 populations from 551 to 2155 m. a.s.l. and *C. palustre* : 285 populations from 212 to 2080 m. a.s.l.; see Figures S5 and S6), ensuring that major climatic and edaphic variation was well represented. This suggests that the relationship between female frequency and broad‐scale environmental conditions is generally weak or absent in the two studied *Cirsium* species, at least for the environmental predictors examined in this study. Environmental correlates of population sex ratios vary considerably among gynodioecious taxa. *Stellaria graminea* (Caryophyllaceae), population sex ratio covaried only with mean annual temperature (Kučera et al. 2021). Although dimorphic populations are frequently associated with drier habitats—consistent with the SDP hypothesis that resource limitation disproportionately reduces hermaphrodite reproduction (Delph 2003; Van Etten and Chang 2009)—empirical support is mixed. Drier habitats favored female establishment in *Wurmbea biglandulosa* (Colchicaceae) by reducing hermaphrodite seed fitness (Vaughton and Ramsey 2004) and in *Lobelia siphilitica* (Campanulaceae) female frequency declined with soil moisture and fruit predation but increased with temperature (Caruso and Case 2007). In contrast, dimorphic and monomorphic *Geranium maculatum* (Geraniaceae) occurred in similar moisture conditions (Van Etten and Chang 2009), and *Cirsium chikushiense* (Asteraceae) showed habitat‐independent variation in female frequencies (Kawakubo 1994). In *Silene acaulis* (Caryophyllaceae), female frequency increases with latitude as reduced solar angles limit cushion microclimatic benefits (Svoen et al. 2019), and Varga and Soulsbury (2020) similarly suggest that thermal stress can enhance female advantage and thereby female frequency. Overall, our results suggest that broad‐scale environmental predictors explain little variation in female frequency in *C. heterophyllum* and *C. palustre* . More broadly, studies of gynodioecious species have reported a diversity of sex‐ratio–environment relationships, indicating that the importance of environmental drivers is context‐dependent and varies among taxa and ecological settings. Further studies across additional *Cirsium* species will be needed to determine whether any general patterns emerge.

## 5 Conclusions

Our study shows that female frequencies differ sharply between the two congeners: the polycarpic *C. heterophyllum* maintains high female frequencies, whereas the monocarpic *C. palustre* exhibits low frequencies. These differences closely mirror species‐level patterns of female advantage, which were strong in *C. heterophyllum* (3.77) but weak in *C. palustre* (1.26). The components contributing to female advantage also differ between the two studied *Cirsium* species and may partly reflect their contrasting life histories. Vegetative traits did not contribute to female advantage in either species. Instead, reproductive traits dominated: achene viability was the primary driver in *C. heterophyllum* , whereas floret production contributed most in *C. palustre* . Although hermaphrodites suffered higher predispersal granivory in both species, granivory susceptibility related traits explained only a small proportion of the female advantage. Environmental predictors played only a limited role in shaping female frequencies; temperature seasonality accounted for a small yet significant fraction of female‐frequency variation in *C. palustre* , and no environmental variables predicted female frequency in *C. heterophyllum* . Taken together, our findings suggest that reproductive output and reproductive efficiency are important contributors to female persistence in the two studied gynodioecious *Cirsium* species, whereas vegetative performance and environmental variation appear to play comparatively limited roles. Differences in female persistence between the studied species may also be partly associated with their contrasting life‐history strategies.

## Author Contributions

**Ashini Dias Mahadura:** formal analysis (lead), investigation (equal), methodology (equal), writing – original draft (lead). **Jakub Šmerda:** investigation (supporting), methodology (supporting). **Tereza Maňáková:** investigation (supporting). **Michaela Kubová:** investigation (supporting). **Zuzana Halamová:** investigation (supporting). **František Zedek:** formal analysis (supporting), writing – review and editing (supporting). **Petr Bureš:** conceptualization (lead), investigation (equal), methodology (lead), project administration (lead), resources (lead), supervision (lead), writing – review and editing (lead).

## Funding

The authors have nothing to report.

## Conflicts of Interest

The authors declare no conflicts of interest.

## Supporting information


**Data S1:** contains the raw data used for all statistical analyses. The file includes a dedicated ReadMe sheet describing the datasets, variables, abbreviations, and measurement units.

**Table S1:** ece374093‐sup‐0002‐TableS1‐S22‐FigureS1‐S8.docx. *Cirsium heterophyllum* female advantage (♀/⚥) at the population and species levels. Mean number of viable achenes per plant (± SE) in female and hermaphrodite individuals for each population (coordinates). The female advantage ratio (♀/⚥) was calculated as the ratio of the mean number of viable achenes per female plant to that per hermaphrodite plant. Species‐level values represent means (± SE) calculated across all populations. *N* indicates the number of individuals per sex.
**Table S2:** *Cirsium palustre* female advantage (♀/⚥) at the population and species levels. Mean number of viable achenes per plant (± SE) in female and hermaphrodite individuals for each population (coordinates). The female advantage ratio (♀/⚥) was calculated as the ratio of the mean number of viable achenes per female plant to that per hermaphrodite plant. Species‐level values represent means (± SE) calculated across all populations. N indicates the number of individuals per sex.
**Table S3:** Differences in plant height between sexual morphs in *C. heterophyllum* and *C. palustre*. Results are from linear mixed‐effects models (LMMs) fitted separately for each species, with Sex (Female vs. Hermaphrodite) as a fixed effect and Locality as a random intercept. Values shown are estimated marginal means contrasts (Female—Hermaphrodite), with standard errors (SE), denominator degrees of freedom (df), t‐values, and Tukey‐adjusted *p*‐values. Sex is denoted as F = Female and H = Hermaphrodite.
**Table S4:** Differences in stem robustness between sexual morphs in *C. heterophyllum* and *C. palustre*. Results are from LMMs fitted separately for each species, with Sex (Female vs. Hermaphrodite) as a fixed effect and Locality as a random intercept. Values shown are estimated marginal means contrasts (Female—Hermaphrodite), with standard errors (SE), denominator degrees of freedom (df), t‐values, and Tukey‐adjusted *p*‐values. Sex is denoted as F = Female and H = Hermaphrodite.
**Table S5:** Differences in florets per plant between sexual morphs in *C. heterophyllum* and *C. palustre*. Results of GLMMs (Poisson) testing differences in the number of florets per plant within each species between sexual morphs (female vs. hermaphrodite). Separate models were fitted for *C. heterophyllum* and *C. palustre*, each including Sex as a fixed effect and Locality as a random intercept. Estimated marginal means (± SE) and Tukey‐adjusted pairwise comparisons are provided. Sex is denoted as F = Female and H = Hermaphrodite.
**Table S6:** Differences in viable achenes per plant between sexual morphs in *C. heterophyllum* and *C. palustre*. Results of GLMMs (Poisson) testing differences in the number of viable achenes per plant within each species between sexual morphs (female vs. hermaphrodite). Separate models were fitted for *C. heterophyllum* and *C. palustre*, each including Sex as a fixed effect and Locality as a random intercept. Estimated marginal means (± SE) and Tukey‐adjusted pairwise comparisons are provided. Sex is denoted as F = Female and H = Hermaphrodite.
**Table S7:** Differences in achene viability (%) between sexual morphs in *C. heterophyllum* and *C. palustre*. Results are from GLMMs (binomial) testing differences in the proportion of viable achenes between females and hermaphrodites within each species. Separate models were fitted for *C. heterophyllum* and *C. palustre*, with Sex as a fixed effect and Locality as a random intercept. Estimates are on the logit scale. Estimated marginal means (± SE) were back‐transformed to percentages, and Tukey‐adjusted pairwise comparisons are provided. A positive estimate indicates higher achene viability in females compared to hermaphrodites. Sex is denoted as F = Female and H = Hermaphrodite.
**Table S8:** Differences in granivores per plant between sexual morphs in *C. heterophyllum* and *C. palustre*. Results of GLMMs (Poisson) testing differences in the number of granivores per plant within each species between sexual morphs (female vs. hermaphrodite). Separate models were fitted for *C. heterophyllum* and *C. palustre*, each including Sex as a fixed effect and Locality as a random intercept. Estimated marginal means (± SE) and Tukey‐adjusted pairwise comparisons are provided. Sex is denoted as F = Female and H = Hermaphrodite.
**Table S9:** *Cirsium heterophyllum* female advantage contributing trait ratio at the population level.
**Table S10:** *Cirsium palustre* female advantage contributing trait ratio at the population level.
**Table S11:** Trait contributions to female advantage. Relative contributions of three sex‐specific trait ratios—florets per plant (F/H), achene viability (%) (F/H), and granivory (H/F)—to female advantage in *C. heterophyllum* and *C. palustre*. Trait importance was quantified using the change in explained variance (Δ*R* ^2^) between the full linear model and reduced models in which each focal trait was removed. The full *R* ^2^ represents the variance explained by the full model, which includes all three traits. Reduced *R* ^2^ represents the variance explained when the focal trait is excluded from the model. Δ*R* ^2^ (Full *R* ^2^ − Reduced *R* ^2^) indicates the variance explained by each trait, and Contribution (%) expresses Δ*R* ^2^ as a proportion of the sum of all Δ*R* ^2^ values for each species. These percentage contributions correspond directly to the proportions displayed in Figure 9 (doughnut charts).
**Table S12:** Differences in capitula per plant between sexual morphs in *C. heterophyllum* and *C. palustre*. Results of GLMMs (Poisson) testing differences in the number of capitula per plant within each species between sexual morphs (female vs. hermaphrodite). Separate models were fitted for *C. heterophyllum* and *C. palustre*, each including Sex as a fixed effect and Locality as a random intercept. Estimated marginal means (± SE) and Tukey‐adjusted pairwise comparisons are provided. Sex is denoted as F = Female and H = Hermaphrodite.
**Table S13:** Stem robustness on florets per plant results of model comparison for *C. heterophyllum*.
**Table S14:** Stem robustness on florets per plant: results of the interactive model for *C. heterophyllum*.
**Table S15:** Stem robustness on florets per plant results of model comparison for *C. palustre*.
**Table S16:** Stem robustness on florets per plant: results of the interactive model for *C. palustre*.
**Table S17:** *Cirsium heterophyllum* means (± SE) of plant height, stem robustness, and granivory at the population level.
**Table S18:** *Cirsium palustre* means (± SE) of plant height, stem robustness, and granivory at the population level.
**Table S19:** Linear regressions testing population‐level environmental predictors of female advantage in *C. heterophyllum* and *C. palustre*.
**Table S20:** Linear regressions testing population‐level environmental predictors of female frequency in *C. heterophyllum* and *C. palustre*.
**Table S21:** Results of single‐predictor weighted least‐squares (WLS) regressions explaining variation in the female frequency of *C. heterophyllum* across populations as a function of 22 environmental predictors. Predictors include 19 bioclimatic variables (BIO1–BIO19) from the CHELSA database and three soil variables (pH in H₂O, total nitrogen content, and gravel volume at 0–5 cm depth) from SoilGrids. The log‐transformed sample size was used as a weighting factor to account for differences in precision among populations. Reported values include standardized regression coefficients (*β*), coefficients of determination (*R* ^2^; proportion of variation explained), raw *p*‐values, and adjusted *p*‐values after Sidák correction for multiple testing based on the effective number of independent predictors (Meff = 7.07; significance threshold *p* < 0.007).
**Table S22:** Results of single‐predictor weighted least‐squares (WLS) regressions explaining variation in the female frequency of *C. palustre* across populations as a function of 22 environmental predictors. Predictors include 19 bioclimatic variables (BIO1–BIO19) from the CHELSA database and three soil variables (pH in H₂O, total nitrogen content, and gravel volume at 0–5 cm depth) from SoilGrids. The log‐transformed sample size was used as a weighting factor to account for differences in precision among populations. Reported values include standardized regression coefficients (*β*), coefficients of determination (*R* ^2^; proportion of variation explained), raw *p*‐values, and adjusted *p*‐values after Sidák correction for multiple testing based on the effective number of independent predictors (Meff = 7.07; significance threshold *p* < 0.007).
**Figure S1:** Comparison of capitula numbers per plant between female and hermaphroditic individuals in polycarpic *Cirsium heterophyllum* (A) and monocarpic *C. palustre* (B). Significant sex differences on generalized linear mixed‐effects models (GLMMs; *p* < 0.05) are shown in red, with the larger sex indicated by “>” between females (F) and hermaphrodites (H). Observed capitula per plant means (± SE) were as follows: *C. heterophyllum*—F = 2.10 ± 0.06, H = 2.18 ± 0.07; *C. palustre*—F = 195 ± 6.55, H = 182 ± 5.75.
**Figure S2:** Relationship between stem robustness and florets per plant in *C. heterophyllum* and *C. palustre*. Florets per plant were analyzed using Poisson generalized linear mixed‐effects models (GLMMs) with locality as a random effect. For both species, the model including the Sex × Robustness interaction provided a significantly better fit than the additive model. In *C. heterophyllum*, inclusion of the interaction improved model fit (ANOVA likelihood‐ratio test, LRT: χ2₁ = 7.38, *p* = 0.007; Table S13). In *C. palustre*, florets per plant showed a highly significant Sex × Robustness interaction (ANOVA LRT: χ2₁ = 788.57, *p* < 0.001; Table S15). Lines indicate model‐predicted relationships between stem robustness and florets per plant for each sex, and shaded areas show 95% confidence intervals; gray points represent individual plants. Note. Across both species, florets per plant scaled positively with stem robustness, indicating that reproductive output increases with overall plant robustness at the individual level (Tables S14 and S16). In both *C. heterophyllum* and *C. palustre*, the significant Sex × Robustness interaction demonstrates that the effect of robustness on reproductive output differs between sexes. Specifically, hermaphrodites showed a slightly stronger increase in florets with rising robustness, whereas females consistently produced more florets overall across the full robustness range.
**Figure S3:** Comparison of plant height (cm) between females (red) and hermaphrodites (blue) across populations of *C. heterophyllum*. Boxplots show the interquartile range, median (thick line), and individual plants (points). Sex differences within each population were tested using Wilcoxon rank‐sum tests, with significant differences indicated by asterisks (*p* < 0.05 = *; *p* < 0.01 = **). According to a two‐way ANOVA, plant height differed strongly among populations (*p* < 0.001).
**Figure S4:** Comparison of plant height (cm) between females (red) and hermaphrodites (blue) across populations of *C. palustre*. Boxplots show the interquartile range, median (thick line), and individual plants (points). Sex differences within each population were tested using Wilcoxon rank‐sum tests, and no significant differences were detected. Based on the two‐way ANOVA, plant height differed significantly among populations (*p* < 0.001).
**Figure S5:** Altitudinal distribution of 126 populations (sample localities) of *C. heterophyllum*.
**Figure S6:** Altitudinal distribution of 126 populations (sample localities) of *C. palustre*.
**Figure S7:** Pearson correlation heat map of the 22 environmental predictors (19 CHELSA bioclimatic variables and 3 soil variables). Variables are ordered by hierarchical clustering of |r| values, and the seven resulting predictor clusters are outlined with black rectangles. Correlation coefficients are shown in the upper triangle, while the lower triangle displays the corresponding color scale (blue = negative, red = positive). This correlation structure formed the basis of the Li and Ji (2005) spectral decomposition, yielding an effective number of independent predictors of Meff = 7.07.
**Figure S8:** Identified granivores to species level. *Tephritis conura* (A). *Larinus sturnus* (B). *Larinus turbinatus* (C). Photo credits: Petr Bureš.

## Data Availability

The data supporting the findings of this study are available as Supporting Information accompanying this article.
