# Supplementary material for: How Do Females Persist? Life History, Environment, and Female Advantage in Gynodioecious Cirsium
Source: Ecol Evol. 2026 Jul 24;16(7):e74093. doi: 10.1002/ece3.74093 (PMC13400990; doi:10.1002/ece3.74093)
Supplement: Supplementary file 2 — Table S1: ece374093‐sup‐0002‐TableS1‐S22‐FigureS1‐S8.docx. Cirsium heterophyllum female advantage (♀/⚥) at the population and species levels. Mean number of viable achenes per plant (± SE) in female and hermaphrodite individuals for each population (coordinates). The female advantage ratio (♀/⚥) was calculated as the ratio of the mean number of viable achenes per female plant to that per hermaphrodite plant. Species‐level values represent means (± SE) calculated across all populations. N indicates the number of individuals per sex. Table S2: Cirsium palustre female advantage (♀/⚥) at the population and species levels. Mean number of viable achenes per plant (± SE) in female and hermaphrodite individuals for each population (coordinates). The female advantage ratio (♀/⚥) was calculated as the ratio of the mean number of viable achenes per female plant to that per hermaphrodite plant. Species‐level values represent means (± SE) calculated across all populations. N indicates the number of individuals per sex. Table S3: Differences in plant height between sexual morphs in C. heterophyllum and C. palustre. Results are from linear mixed‐effects models (LMMs) fitted separately for each species, with Sex (Female vs. Hermaphrodite) as a fixed effect and Locality as a random intercept. Values shown are estimated marginal means contrasts (Female—Hermaphrodite), with standard errors (SE), denominator degrees of freedom (df), t‐values, and Tukey‐adjusted p‐values. Sex is denoted as F = Female and H = Hermaphrodite. Table S4: Differences in stem robustness between sexual morphs in C. heterophyllum and C. palustre. Results are from LMMs fitted separately for each species, with Sex (Female vs. Hermaphrodite) as a fixed effect and Locality as a random intercept. Values shown are estimated marginal means contrasts (Female—Hermaphrodite), with standard errors (SE), denominator degrees of freedom (df), t‐values, and Tukey‐adjusted p‐values. Sex is denoted as F = Female and H = Hermaphr [file ECE3-16-e74093-s002.docx]

**Supporting Information: Supplementary tables 1–22 and Supplementary figures S1–S8**

**Supplementary Table 1.** *Cirsium heterophyllum* female advantage (♀/⚥) at the population and species levels. Mean number of viable achenes per plant (± SE) in female and hermaphrodite individuals for each population (coordinates). The female advantage ratio (♀/⚥) was calculated as the ratio of the mean number of viable achenes per female plant to that per hermaphrodite plant. Species-level values represent means (± SE) calculated across all populations. *N* indicates the number of individuals per sex.

| **Coordinates** | **N (♀)** | **Mean Viable Achenes/Plant (♀) (± SE)** | **N (**⚥**)** | **Mean Viable Achenes/Plant (**⚥**) (± SE)** | **Female Advantage Ratio (♀/**⚥**)** | **Female Frequency (%)** |
| --- | --- | --- | --- | --- | --- | --- |
| 49°39'29.3"N, 15°54'11.7"E | 20 | 35.52 ± 7.92 | 20 | 13.5 ± 6.78 | 2.63 | 60.61 |
| 49°38'14.3"N, 15°51'52.7"E | 20 | 67.78 ± 21.48 | 20 | 11.68 ± 4.93 | 5.80 | 52.80 |
| 49°40'56.7"N, 15°57'41.4"E | 18 | 10.05 ± 5.26 | 22 | 1.56 ± 1.32 | 6.46 | 44.10 |
| 49°42'11.3"N, 15°55'55.1"E | 20 | 221.01 ± 44.74 | 20 | 47.97 ± 17.42 | 4.61 | 55.98 |
| 49°43'10.2"N, 15°59'53.7"E | 20 | 57.33 ± 15.95 | 20 | 10.62 ± 5.97 | 5.40 | 60.47 |
| 49°37'48.0"N, 16°05'09.1"E | 20 | 57.94 ± 14.03 | 20 | 11.88 ± 7.49 | 4.88 | 52.45 |
| 49°40'34.0"N, 16°05'15.5"E | 20 | 14.67 ± 6.68 | 20 | 6.75 ± 3.16 | 2.17 | 52.84 |
| 49°38'59.5"N, 16°02'57.1"E | 20 | 131.75 ± 15.78 | 20 | 34.36 ± 12.31 | 3.83 | 48.39 |
| 50°44'37.5"N, 15°49'24.4"E | 20 | 41.08 ± 7.94 | 20 | 14.61 ± 4.63 | 2.81 | 61.54 |
| 50°44'17.6"N, 15°48'11.1"E | 20 | 108.91 ± 19.21 | 20 | 45.63 ± 12.12 | 2.39 | 57.80 |
| 50°44'09.9"N, 15°47'56.0"E | 20 | 61.89 ± 15.33 | 20 | 14.21 ± 4.48 | 4.36 | 55.53 |
| 50°41'13.6"N, 15°43'49.2"E | 20 | 92.73 ± 19.55 | 20 | 65.53 ± 16.62 | 1.42 | 47.71 |
| 50°40'54.4"N, 15°43'26.7"E | 20 | 34.58 ± 14.27 | 20 | 15.30 ± 7.78 | 2.26 | 57.65 |
| **Species level** | **258** | **71.94 ± 56.94** | **262** | **22.58 ± 19.36** | **3.77** | **54.45** |

**Supplementary Table 2.** *Cirsium palustre* female advantage (♀/⚥) at the population and species levels. Mean number of viable achenes per plant (± SE) in female and hermaphrodite individuals for each population (coordinates). The female advantage ratio (♀/⚥) was calculated as the ratio of the mean number of viable achenes per female plant to that per hermaphrodite plant. Species-level values represent means (± SE) calculated across all populations. *N* indicates the number of individuals per sex.

| **Coordinates** | **N (♀)** | **Mean Viable Achenes/Plant (♀) (± SE)** | **N (**⚥**)** | **Mean Viable Achenes/Plant (**⚥**) (± SE)** | **Female Advantage Ratio (♀/**⚥**)** | **Female Frequency (%)** |
| --- | --- | --- | --- | --- | --- | --- |
| 49°15'15.5"N, 16°18'40.0"E | 9 | 4363.83 ± 1254.94 | 9 | 2494.83 ± 1081.85 | 1.75 | 26.09 |
| 49°15'03.4"N, 16°16'19.5"E | 13 | 3232.02 ± 872.1 | 13 | 2235.58 ± 626.85 | 1.45 | 8.66 |
| 49°21'19.8"N, 16°07'28.9"E | 11 | 9940.66 ± 1522.88 | 11 | 8813.7 ± 1745.92 | 1.13 | 19.63 |
| 49°11'45.8"N, 15°37'39.1"E | 20 | 6320.62 ± 884.38 | 20 | 4858.42 ± 1010.75 | 1.30 | 16.54 |
| 49°10'26.5"N, 15°38'05.9"E | 18 | 7594.47 ± 1238.63 | 18 | 6557.76 ± 895.63 | 1.16 | 29.78 |
| 49°28'23.8"N, 16°26'47.9"E | 17 | 8859.01 ± 2000.62 | 17 | 9047.07 ± 1634.54 | 0.98 | 25.37 |
| 49°29'03.4"N, 16°24'36.8"E | 17 | 4131.38 ± 1398.65 | 17 | 3191.97 ± 935.79 | 1.29 | 26.74 |
| 49°35'50.1"N, 16°15'24.8"E | 20 | 8307.41 ± 1198.72 | 20 | 6472.66 ± 814.93 | 1.28 | 22.81 |
| 49°43'53.1"N, 15°55'52.5"E | 15 | 10028.28 ± 1015.72 | 15 | 13025.67 ± 2704.73 | 0.77 | 62.07 |
| 49°43'01.9"N, 15°55'58.1"E | 20 | 9420.08 ± 1685.76 | 20 | 8527.09 ± 1491.33 | 1.11 | 13.95 |
| 49°27'48.2"N 16°01'36.0"E | 20 | 8690.79 ± 1049.2 | 20 | 5402.24 ± 598.71 | 1.61 | 46.69 |
| 49°35'08.0"N, 16°06'53.1"E | 20 | 10904.83 ± 767.32 | 20 | 10607.94 ± 1047.98 | 1.03 | 24.14 |
| 49°43'26.6"N, 16°00'32.0"E | 14 | 7581.45 ± 1071.35 | 14 | 5840.18 ± 1047.33 | 1.30 | 14.36 |
| 49°37'01.7"N, 16°00'25.3"E | 20 | 6518.48 ± 1206.88 | 20 | 5012.05 ± 664.12 | 1.30 | 50.99 |
| 49°26'34.5"N, 16°02'51.9"E | 18 | 8513.82 ± 1460.6 | 18 | 6187.19 ± 805.07 | 1.38 | 31.29 |
| 49°47'01.8"N, 16°10'02.1"E | 12 | 7457.00 ± 1608.44 | 12 | 5624.81 ± 1268.49 | 1.33 | 17.59 |
| **Species level** | **264** | **7616.51 ± 2223.01** | **264** | **6493.70 ± 2921.14** | **1.26** | **27.29** |

**Supplementary Table 3. Differences in plant height between sexual morphs in *C. heterophyllum* and *C. palustre*.** Results are from linear mixed-effects models (LMMs) fitted separately for each species, with Sex (Female vs. Hermaphrodite) as a fixed effect and Locality as a random intercept. Values shown are estimated marginal means contrasts (Female – Hermaphrodite), with standard errors (SE), denominator degrees of freedom (df), t-values, and Tukey-adjusted p-values. Sex is denoted as F = Female and H = Hermaphrodite.

| **Species: Effect / Contrast** | **Estimate** | **SE** | **df** | ***t / F*** | ***p*-value** |
| --- | --- | --- | --- | --- | --- |
| *C. heterophyllum*: Sex (F vs H) | –0.45 | 1.72 | 1017.6 | –0.26 | 0.72 |
| *C. palustre*: Sex (F vs H) | –2.10 | 1.71 | 1017.4 | –1.02 | 0.31 |

**Supplementary Table 4. Differences in stem robustness between sexual morphs in *C. heterophyllum* and *C. palustre*.** Results are from LMMs fitted separately for each species, with Sex (Female vs. Hermaphrodite) as a fixed effect and Locality as a random intercept. Values shown are estimated marginal means contrasts (Female – Hermaphrodite), with standard errors (SE), denominator degrees of freedom (df), t-values, and Tukey-adjusted p-values. Sex is denoted as F = Female and H = Hermaphrodite.

| **Species: Effect / Contrast** | **Estimate** | **SE** | **df** | ***t / F*** | ***p*-value** |
| --- | --- | --- | --- | --- | --- |
| *C. heterophyllum*: Sex (F vs H) | –0.00285 | 0.00614 | 1018 | –2.14 | 0.034 |
| *C. palustre*: Sex (F vs H) | 0.00059 | 0.00609 | 1017 | 0.07 | 0.944 |

**Supplementary Table 5. Differences in florets per plant between sexual morphs in *C. heterophyllum* and *C. palustre*.** Results of GLMMs (Poisson) testing differences in the number of florets per plant within each species between sexual morphs (female vs. hermaphrodite). Separate models were fitted for *C. heterophyllum* and *C. palustre*, each including Sex as a fixed effect and Locality as a random intercept. Estimated marginal means (± SE) and Tukey-adjusted pairwise comparisons are provided. Sex is denoted as F = Female and H = Hermaphrodite.

| **Species: Effect / Contrast** | **Estimate** | **SE** | **df** | ***z / χ²*** | ***p*-value** |
| --- | --- | --- | --- | --- | --- |
| *C. heterophyllum*: Sex (F vs H) | 0.08344 | 0.00499 | 517 | 16.71 | <0.001 |
| *C. palustre*: Sex (F vs H) | 0.09285 | 0.00075 | 525 | 123.7 | <0.001 |

**Supplementary Table 6. Differences in viable achenes per plant between sexual morphs in *C. heterophyllum* and *C. palustre*.** Results of GLMMs (Poisson) testing differences in the number of viable achenes per plant within each species between sexual morphs (female vs. hermaphrodite). Separate models were fitted for *C. heterophyllum* and *C. palustre*, each including Sex as a fixed effect and Locality as a random intercept. Estimated marginal means (± SE) and Tukey-adjusted pairwise comparisons are provided. Sex is denoted as F = Female and H = Hermaphrodite.

| **Species: Effect / Contrast** | **Estimate** | **SE** | **df** | ***z / χ²*** | ***p*-value** |
| --- | --- | --- | --- | --- | --- |
| *C. heterophyllum*: Sex (F vs H) | 1.15912 | 0.01496 | 517 | 77.49 | <0.001 |
| *C. palustre*: Sex (F vs H) | 0.15961 | 0.00103 | 525 | 155.05 | <0.001 |

**Supplementary Table 7. Differences in achene viability (%) between sexual morphs in *C. heterophyllum* and *C. palustre*.** Results are from GLMMs (binomial) testing differences in the proportion of viable achenes between females and hermaphrodites within each species. Separate models were fitted for *C. heterophyllum* and *C. palustre*, with Sex as a fixed effect and Locality as a random intercept. Estimates are on the logit scale. Estimated marginal means (± SE) were back-transformed to percentages, and Tukey-adjusted pairwise comparisons are provided. A positive estimate indicates higher achene viability in females compared to hermaphrodites. Sex is denoted as F = Female and H = Hermaphrodite.

| **Species: Effect / Contrast** | **Estimate** | **SE** | **df** | ***z / χ²*** | ***p*-value** |
| --- | --- | --- | --- | --- | --- |
| *C. heterophyllum*: Sex (F vs H) | 1.30632 | 0.01677 | 514 | 77.91 | <0.001 |
| *C. palustre*: Sex (F vs H) | 0.11951 | 0.00358 | 525 | 33.36 | <0.001 |

**Supplementary Table 8. Differences in granivores per plant between sexual morphs in *C. heterophyllum* and *C. palustre*.** Results of GLMMs (Poisson) testing differences in the number of granivores per plant within each species between sexual morphs (female vs. hermaphrodite). Separate models were fitted for *C. heterophyllum* and *C. palustre*, each including Sex as a fixed effect and Locality as a random intercept. Estimated marginal means (± SE) and Tukey-adjusted pairwise comparisons are provided. Sex is denoted as F = Female and H = Hermaphrodite.

| **Species: Effect / Contrast** | **Estimate** | **SE** | **df** | ***z / χ²*** | ***p*-value** |
| --- | --- | --- | --- | --- | --- |
| *C. heterophyllum*: Sex (F vs H) | - 0.28571 | 0.06189 | 517 | -4.62 | <0.001 |
| *C. palustre*: Sex (F vs H) | -0.28988 | 0.06178 | 525 | -4.69 | <0.001 |

**Supplementary Table 9.** *Cirsium heterophyllum* female advantage contributing trait ratio at the population level.

| **Coordinates** | **Florets Ratio (♀/**⚥**)** | **Achene Viability Ratio (♀/**⚥**)** | | | | **Granivory Ratio (**⚥**/♀)** |
| --- | --- | --- | --- | --- | --- | --- |
| 49°39'29.3"N, 15°54'11.7"E | 0.96 | | 3.52 | | 0.72 | |
| 49°38'14.3"N, 15°51'52.7"E | 0.96 | | 7.27 | 1.91 | | |
| 49°40'56.7"N, 15°57'41.4"E | 1.31 | | 6.52 | 0.69 | | |
| 49°42'11.3"N, 15°55'55.1"E | 1.56 | | 3.79 | 0.90 | | |
| 49°43'10.2"N, 15°59'53.7"E | 1.17 | | 5.43 | 2.20 | | |
| 49°37'48.0"N, 16°05'09.1"E | 0.92 | | 2.86 | 1.28 | | |
| 49°40'34.0"N, 16°05'15.5"E | 0.87 | | 2.56 | 1.52 | | |
| 49°38'59.5"N, 16°02'57.1"E | 1.29 | | 3.69 | 3.38 | | |
| 50°44'37.5"N, 15°49'24.4"E | 1.36 | | 2.82 | 0.65 | | |
| 50°44'17.6"N, 15°48'11.1"E | 1.25 | | 1.88 | 3.63 | | |
| 50°44'09.9"N, 15°47'56.0"E | 1.26 | | 3.48 | 2.13 | | |
| 50°41'13.6"N, 15°43'49.2"E | 0.83 | | 1.84 | 4.36 | | |
| 50°40'54.4"N, 15°43'26.7"E | 0.83 | | 2.39 | 4.10 | | |

**Supplementary Table 10.** *Cirsium palustre* female advantage contributing trait ratio at the population level.

| **Coordinates** | **Florets Ratio (♀/**⚥**)** | **Achene Viability Ratio (♀/**⚥**)** | **Granivory Ratio (**⚥**/♀)** |
| --- | --- | --- | --- |
| 49°15'15.5"N, 16°18'40.0"E | 0.95 | 1.32 | 11.12 |
| 49°15'03.4"N, 16°16'19.5"E | 1.02 | 0.90 | 1.07 |
| 49°21'19.8"N, 16°07'28.9"E | 1.02 | 0.99 | 1.37 |
| 49°11'45.8"N, 15°37'39.1"E | 1.13 | 1.01 | 1.26 |
| 49°10'26.5"N, 15°38'05.9"E | 1.36 | 1.01 | 1.94 |
| 49°28'23.8"N, 16°26'47.9"E | 0.99 | 0.90 | 2.31 |
| 49°29'03.4"N, 16°24'36.8"E | 1.20 | 1.11 | 13.76 |
| 49°35'50.1"N, 16°15'24.8"E | 1.09 | 1.07 | 1.37 |
| 49°43'53.1"N, 15°55'52.5"E | 0.99 | 1.05 | 1.27 |
| 49°43'01.9"N, 15°55'58.1"E | 1.03 | 1.01 | 1.57 |
| 49°27'48.2"N 16°01'36.0"E | 1.30 | 1.00 | 0.58 |
| 49°35'08.0"N, 16°06'53.1"E | 1.04 | 0.99 | 0.74 |
| 49°43'26.6"N, 16°00'32.0"E | 0.99 | 1.01 | 2.06 |
| 49°37'01.7"N, 16°00'25.3"E | 1.03 | 1.01 | 1.89 |
| 49°26'34.5"N, 16°02'51.9"E | 1.06 | 1.01 | 5.24 |
| 49°47'01.8"N, 16°10'02.1"E | 1.35 | 0.89 | 1.11 |

**Supplementary Table 11.** Trait contributions to female advantage. Relative contributions of three sex-specific trait ratios—florets per plant (F/H), achene viability (%) (F/H), and granivory (H/F)—to female advantage in *C. heterophyllum* and *C. palustre*. Trait importance was quantified using the change in explained variance (ΔR²) between the full linear model and reduced models in which each focal trait was removed. The full R² represents the variance explained by the full model, which includes all three traits. Reduced R² represents the variance explained when the focal trait is excluded from the model. ΔR² (Full R² − Reduced R²) indicates the variance explained by each trait, and Contribution (%) expresses ΔR² as a proportion of the sum of all ΔR² values for each species. These percentage contributions correspond directly to the proportions displayed in Figure 9 (doughnut charts).

| **Species** | **Trait** | **Full R²** | **Reduced R²** | **ΔR²** | **Contribution (%)** |
| --- | --- | --- | --- | --- | --- |
| *C. heterophyllum* | Florets | 0.801 | 0.767 | 0.034 | 6.1 |
|  | Achene viability | 0.801 | 0.286 | 0.515 | 92.9 |
|  | Granivory | 0.801 | 0.796 | 0.005 | 1 |
| *C. palustre* | Florets | 0.255 | 0.181 | 0.074 | 49.6 |
|  | Achene viability | 0.255 | 0.188 | 0.067 | 44.6 |
|  | Granivory | 0.255 | 0.246 | 0.009 | 5.8 |

**Supplementary Table 12. Differences in capitula per plant between sexual morphs in *C. heterophyllum* and *C. palustre*.** Results of GLMMs (Poisson) testing differences in the number of capitula per plant within each species between sexual morphs (female vs. hermaphrodite). Separate models were fitted for *C. heterophyllum* and *C. palustre*, each including Sex as a fixed effect and Locality as a random intercept. Estimated marginal means (± SE) and Tukey-adjusted pairwise comparisons are provided. Sex is denoted as F = Female and H = Hermaphrodite.

| **Species: Effect / Contrast** | **Estimate** | **SE** | **df** | ***z / χ²*** | ***p*-value** |
| --- | --- | --- | --- | --- | --- |
| *C. heterophyllum*: Sex (F vs H) | -0.034 | 0.060 | 517 | -0.572 | 0.567 |
| *C. palustre*: Sex (F vs H) | 0.066 | 0.006 | 525 | 10.430 | <0.001 |

**Supplementary Table 13**. Stem robustness on florets per plant results of model comparison for *C. heterophyllum.*

| **Model** | **Formula** | **AIC** | ***χ²*** | ***p-*value** |
| --- | --- | --- | --- | --- |
| Additive | Florets ~ Sex + Robustness + (1 \| Locality) | 20914 |  |  |
| Interactive | Florets ~ Sex * Robustness + (1 \| Locality) | 20909 | 7.38 | 0.007 |

**Supplementary Table 14**. Stem robustness on florets per plant: results of the interactive model for *C. heterophyllum*.

| **Effect** | **Estimate** | **SE** | ***z*** | ***p*-value** |
| --- | --- | --- | --- | --- |
| Intercept | 5.752 | 0.041 | 139.81 | <0.001 |
| Sex (F vs H) | 0.143 | 0.005 | 27.51 | <0.001 |
| Robustness (z-scaled) | 0.259 | 0.003 | 76.28 | <0.001 |
| Sex x Robustness | -0.012 | 0.004 | -2.72 | 0.007 |

**Supplementary Table 15.** Stem robustness on florets per plant results of model comparison for *Cirsium palustre.*

| **Model** | **Formula** | **AIC** | ***χ²*** | ***p-*value** |
| --- | --- | --- | --- | --- |
| Additive | Florets ~ Sex + Robustness + (1 \| Locality) | 701315 |  |  |
| Interactive | Florets ~ Sex * Robustness + (1 \| Locality) | 700528 | 788.57 | <0.001 |

**Supplementary Table 16.** Stem robustness on florets per plant: results of the interactive model for *C. palustre.*

| **Effect** | **Estimate** | **SE** | ***z*** | ***p*-value** |
| --- | --- | --- | --- | --- |
| Intercept | 9.446 | 0.032 | 291.24 | <0.001 |
| Sex (F vs H) | 0.082 | 0.001 | 102.59 | <0.001 |
| Robustness (z-scaled) | 0.379 | 0.0004 | 887.07 | <0.001 |
| Sex × Robustness | -0.018 | 0.0006 | -28.09 | <0.001 |

**Supplementary Table 17.** *Cirsium heterophyllum* means (± SE) of plant height, stem robustness, and granivory at the population level.

| **Population** | **Coordinates** | **Mean Height** | **Mean Robustness** | **Mean Granivory** |
| --- | --- | --- | --- | --- |
| 1 | 49°39'29.3"N, 15°54'11.7"E | 108.98 ± 2.48 | 0.0621 ± 0.0032 | 4.90 ± 0.49 |
| 2 | 49°38'14.3"N, 15°51'52.7"E | 92.98 ± 2.92 | 0.0334 ± 0.0021 | 0.68 ± 0.26 |
| 3 | 49°40'56.7"N, 15°57'41.4"E | 79.83 ± 2.79 | 0.0488 ± 0.0028 | 2.12 ± 0.48 |
| 4 | 49°42'11.3"N, 15°55'55.1"E | 86.33 ± 1.79 | 0.0450 ± 0.0027 | 1.72 ± 0.36 |
| 5 | 49°43'10.2"N, 15°59'53.7"E | 106.00 ± 2.53 | 0.0506 ± 0.0029 | 2.41 ± 0.47 |
| 6 | 49°37'48.0"N, 16°05'09.1"E | 108.65 ± 1.88 | 0.0475 ± 0.0025 | 4.92 ± 0.77 |
| 7 | 49°40'34.0"N, 16°05'15.5"E | 94.53 ± 2.00 | 0.0499 ± 0.0030 | 1.89 ± 0.54 |
| 8 | 49°38'59.5"N, 16°02'57.1"E | 102.85 ± 2.17 | 0.0432 ± 0.0024 | 1.68 ± 0.37 |
| 9 | 50°44'37.5"N, 15°49'24.4"E | 101.78 ± 2.18 | 0.0474 ± 0.0023 | 1.95 ± 0.30 |
| 10 | 50°44'17.6"N, 15°48'11.1"E | 106.18 ± 2.12 | 0.0429 ± 0.0016 | 1.91 ± 0.49 |
| 11 | 50°44'09.9"N, 15°47'56.0"E | 99.75 ± 2.42 | 0.0421 ± 0.0014 | 1.15 ± 0.22 |
| 12 | 50°41'13.6"N, 15°43'49.2"E | 98.48 ± 1.89 | 0.0480 ± 0.0022 | 0.90 ± 0.28 |
| 13 | 50°40'54.4"N, 15°43'26.7"E | 97.28 ± 2.62 | 0.0315 ± 0.0016 | 0.25 ± 0.15 |

**Supplementary Table 18.** *Cirsium palustre* means (± SE) of plant height, stem robustness, and granivory at the population level.

| **Population** | **Coordinates** | **Mean Height** | **Mean Robustness** | **Mean Granivory** |
| --- | --- | --- | --- | --- |
| 1 | 49°15'15.5"N, 16°18'40.0"E | 162.76 ± 5.21 | 0.1805 ± 0.0192 | 0.87 ± 0.43 |
| 2 | 49°15'03.4"N, 16°16'19.5"E | 164.75 ± 5.07 | 0.1442 ± 0.0096 | 0.95 ± 0.33 |
| 3 | 49°21'19.8"N, 16°07'28.9"E | 175.10 ± 5.09 | 0.2987 ± 0.0334 | 1.34 ± 0.40 |
| 4 | 49°11'45.8"N, 15°37'39.1"E | 183.56 ± 4.82 | 0.2146 ± 0.0162 | 2.70 ± 0.53 |
| 5 | 49°10'26.5"N, 15°38'05.9"E | 185.43 ± 3.57 | 0.2478 ± 0.0138 | 2.38 ± 0.59 |
| 6 | 49°28'23.8"N, 16°26'47.9"E | 173.35 ± 3.94 | 0.2616 ± 0.0238 | 1.89 ± 0.56 |
| 7 | 49°29'03.4"N, 16°24'36.8"E | 177.84 ± 5.36 | 0.2445 ± 0.0239 | 0.51 ± 0.30 |
| 8 | 49°35'50.1"N, 16°15'24.8"E | 186.28 ± 4.69 | 0.2361 ± 0.0121 | 1.74 ± 0.38 |
| 9 | 49°43'53.1"N, 15°55'52.5"E | 163.30 ± 4.02 | 0.2511 ± 0.0160 | 3.19 ± 0.43 |
| 10 | 49°43'01.9"N, 15°55'58.1"E | 161.89 ± 3.79 | 0.2537 ± 0.0192 | 2.50 ± 0.37 |
| 11 | 49°27'48.2"N 16°01'36.0"E | 168.13 ± 2.66 | 0.2527 ± 0.0119 | 1.97 ± 0.36 |
| 12 | 49°35'08.0"N, 16°06'53.1"E | 178.85 ± 3.12 | 0.2523 ± 0.0118 | 3.29 ± 0.62 |
| 13 | 49°43'26.6"N, 16°00'32.0"E | 165.52 ± 3.82 | 0.1926 ± 0.0110 | 1.38 ± 0.42 |
| 14 | 49°37'01.7"N, 16°00'25.3"E | 178.19 ± 2.70 | 0.1665 ± 0.0106 | 0.56 ± 0.20 |
| 15 | 49°26'34.5"N, 16°02'51.9"E | 163.14 ± 3.20 | 0.2230 ± 0.0169 | 0.67 ± 0.18 |
| 16 | 49°47'01.8"N, 16°10'02.1"E | 186.83 ± 4.94 | 0.2138 ± 0.0138 | 7.04 ± 1.27 |

**Supplementary Table 19.** Linear regressions testing population-level environmental predictors of female advantage in *C. heterophyllum and C. palustre.*

| **Species** | **Predictor** | ***β*** | ***p*** | **Adjusted *R²*** |
| --- | --- | --- | --- | --- |
| *C. heterophyllum* | Mean height | –0.071 | 0.19 | 0.070 |
|  | Mean robustness | –23.190 | 0.72 | –0.070 |
|  | Mean granivory | 0.127 | 0.71 | –0.080 |
| *C. palustre* | Mean height | –0.003 | 0.64 | –0.054 |
|  | Mean robustness | –3.220 | 0.03 | 0.244 |
|  | Mean granivory | –0.041 | 0.30 | 0.010 |

**Supplementary Table 20.** Linear regressions testing population-level environmental predictors of female frequency in *C. heterophyllum and C. palustre.*

| **Species** | **Predictor** | ***β*** | ***p*** | **Adjusted *R²*** |
| --- | --- | --- | --- | --- |
| *C. heterophyllum* | Mean height | 0.332 | 0.06 | 0.221 |
|  | Mean robustness | 82.930 | 0.70 | -0.076 |
|  | Mean granivory | 0.723 | 0.53 | -0.052 |
| *C. palustre* | Mean height | -0.195 | 0.65 | -0.055 |
|  | Mean robustness | 47.610 | 0.63 | -0.053 |
|  | Mean granivory | -0.743 | 0.76 | -0.064 |

**Supplementary Table 21.** Results of single-predictor weighted least-squares (WLS) regressions explaining variation in the female frequency of *C. heterophyllum* across populations as a function of 22 environmental predictors. Predictors include 19 bioclimatic variables (BIO1–BIO19) from the CHELSA database and three soil variables (pH in H₂O, total nitrogen content, and gravel volume at 0–5 cm depth) from SoilGrids. The log-transformed sample size was used as a weighting factor to account for differences in precision among populations. Reported values include standardized regression coefficients (*β*), coefficients of determination (R²; proportion of variation explained), raw *p*-values, and adjusted *p*-values after Sidák correction for multiple testing based on the effective number of independent predictors (Meff = 7.07; significance threshold *p* < 0.007).

| **Predictor** | **Explanation of the environmental predictor** | ***β*** | **R^2^** | **Raw *p*** | **Adj *p*** |
| --- | --- | --- | --- | --- | --- |
| BIO1 | Mean annual air temperature | -0.1597 | 0.0232 | 0.089 | 0.628 |
| BIO2 | Mean diurnal air temperature range | 0.5428 | 0.0165 | 0.151 | 1.000 |
| BIO3 | Isothermality | 0.1587 | 0.0097 | 0.273 | 1.000 |
| BIO4 | Temperature seasonality | 0.0000 | 0.0000 | 0.996 | 1.000 |
| BIO5 | Mean daily maximum air temperature of the warmest month | -0.1507 | 0.0221 | 0.096 | 0.682 |
| BIO6 | Mean daily minimum air temperature of the coldest month | -0.1536 | 0.0293 | 0.055 | 0.392 |
| BIO7 | Annual range of air temperature | 0.3242 | 0.0145 | 0.180 | 1.000 |
| BIO8 | Mean daily air temperatures of the wettest quarter | -0.0358 | 0.0043 | 0.467 | 1.000 |
| BIO9 | Mean daily air temperatures of the driest quarter | -0.0927 | 0.0235 | 0.087 | 0.612 |
| BIO10 | Mean daily air temperatures of the warmest quarter | -0.1455 | 0.0220 | 0.098 | 0.691 |
| BIO11 | Mean daily air temperatures of the coldest quarter | -0.2005 | 0.0306 | 0.050 | 0.353 |
| BIO12 | Annual precipitation amount | 0.0192 | 0.0340 | 0.039 | 0.275 |
| BIO13 | Precipitation amount of the wettest month | 0.1226 | 0.0265 | 0.069 | 0.486 |
| BIO14 | Precipitation amount of the driest month | 0.1743 | 0.0120 | 0.221 | 1.000 |
| BIO15 | Precipitation seasonality | -0.0308 | 0.0002 | 0.878 | 1.000 |
| BIO16 | Mean monthly precipitation amount of the wettest quarter | 0.0465 | 0.0310 | 0.049 | 0.345 |
| BIO17 | Mean monthly precipitation amount of the driest quarter | 0.0484 | 0.0089 | 0.294 | 1.000 |
| BIO18 | Mean monthly precipitation amount of the warmest quarter | 0.0420 | 0.0262 | 0.070 | 0.497 |
| BIO19 | Mean monthly precipitation amount of the coldest quarter | 0.0321 | 0.0059 | 0.391 | 1.000 |
| pH H_2_O | pH in soil | -6.3406 | 0.0198 | 0.116 | 0.823 |
| Gravel | Gravel volume (%) | 0.6178 | 0.0137 | 0.192 | 1.000 |
| Nitrogen | Total nitrogen content (g per kg) | 3.1674 | 0.0320 | 0.045 | 0.317 |

**Supplementary Table 22.** Results of single-predictor weighted least-squares (WLS) regressions explaining variation in the female frequency of *C. palustre* across populations as a function of 22 environmental predictors. Predictors include 19 bioclimatic variables (BIO1–BIO19) from the CHELSA database and three soil variables (pH in H₂O, total nitrogen content, and gravel volume at 0–5 cm depth) from SoilGrids. The log-transformed sample size was used as a weighting factor to account for differences in precision among populations. Reported values include standardized regression coefficients (*β*), coefficients of determination (R²; proportion of variation explained), raw *p*-values, and adjusted *p*-values after Sidák correction for multiple testing based on the effective number of independent predictors (Meff = 7.07; significance threshold *p* < 0.007).

| **Predictor** | **Explanation of the environmental predictor** | ***β*** | **R^2^** | **Raw *p*** | **Adj *p*** |
| --- | --- | --- | --- | --- | --- |
| BIO1 | Mean annual air temperature | 0.0654 | 0.0056 | 0.209 | 1.000 |
| BIO2 | Mean diurnal air temperature range | -0.2700 | 0.0048 | 0.242 | 1.000 |
| BIO3 | Isothermality | -0.2357 | 0.0255 | 0.007 | 0.049 |
| **BIO4** | **Temperature seasonality** | **0.0225** | **0.0651** | **<0.001** | **<0.001** |
| BIO5 | Mean daily maximum air temperature of the warmest month | 0.0786 | 0.0086 | 0.119 | 0.838 |
| BIO6 | Mean daily minimum air temperature of the coldest month | 0.0364 | 0.0024 | 0.414 | 1.000 |
| BIO7 | Annual range of air temperature | 0.2286 | 0.0097 | 0.096 | 0.682 |
| BIO8 | Mean daily air temperatures of the wettest quarter | 0.0854 | 0.0315 | 0.003 | 0.019 |
| BIO9 | Mean daily air temperatures of the driest quarter | -0.0485 | 0.0071 | 0.156 | 1.000 |
| BIO10 | Mean daily air temperatures of the warmest quarter | 0.0724 | 0.0076 | 0.141 | 0.995 |
| BIO11 | Mean daily air temperatures of the coldest quarter | 0.0287 | 0.0010 | 0.597 | 1.000 |
| BIO12 | Annual precipitation amount | -0.0009 | 0.0002 | 0.795 | 1.000 |
| BIO13 | Precipitation amount of the wettest month | 0.0231 | 0.0024 | 0.412 | 1.000 |
| BIO14 | Precipitation amount of the driest month | -0.0681 | 0.0041 | 0.279 | 1.000 |
| BIO15 | Precipitation seasonality | 0.2438 | 0.0168 | 0.029 | 0.204 |
| BIO16 | Mean monthly precipitation amount of the wettest quarter | 0.0089 | 0.0028 | 0.374 | 1.000 |
| BIO17 | Mean monthly precipitation amount of the driest quarter | -0.0204 | 0.0036 | 0.314 | 1.000 |
| BIO18 | Mean monthly precipitation amount of the warmest quarter | 0.0111 | 0.0038 | 0.302 | 1.000 |
| BIO19 | Mean monthly precipitation amount of the coldest quarter | -0.0219 | 0.0062 | 0.184 | 1.000 |
| pH H_2_O | pH in soil | -1.8362 | 0.0014 | 0.535 | 1.000 |
| Gravel | Gravel volume (%) | 0.8602 | 0.0231 | 0.010 | 0.072 |
| Nitrogen | Total nitrogen content (g per kg) | -1.8642 | 0.0178 | 0.024 | 0.173 |

**
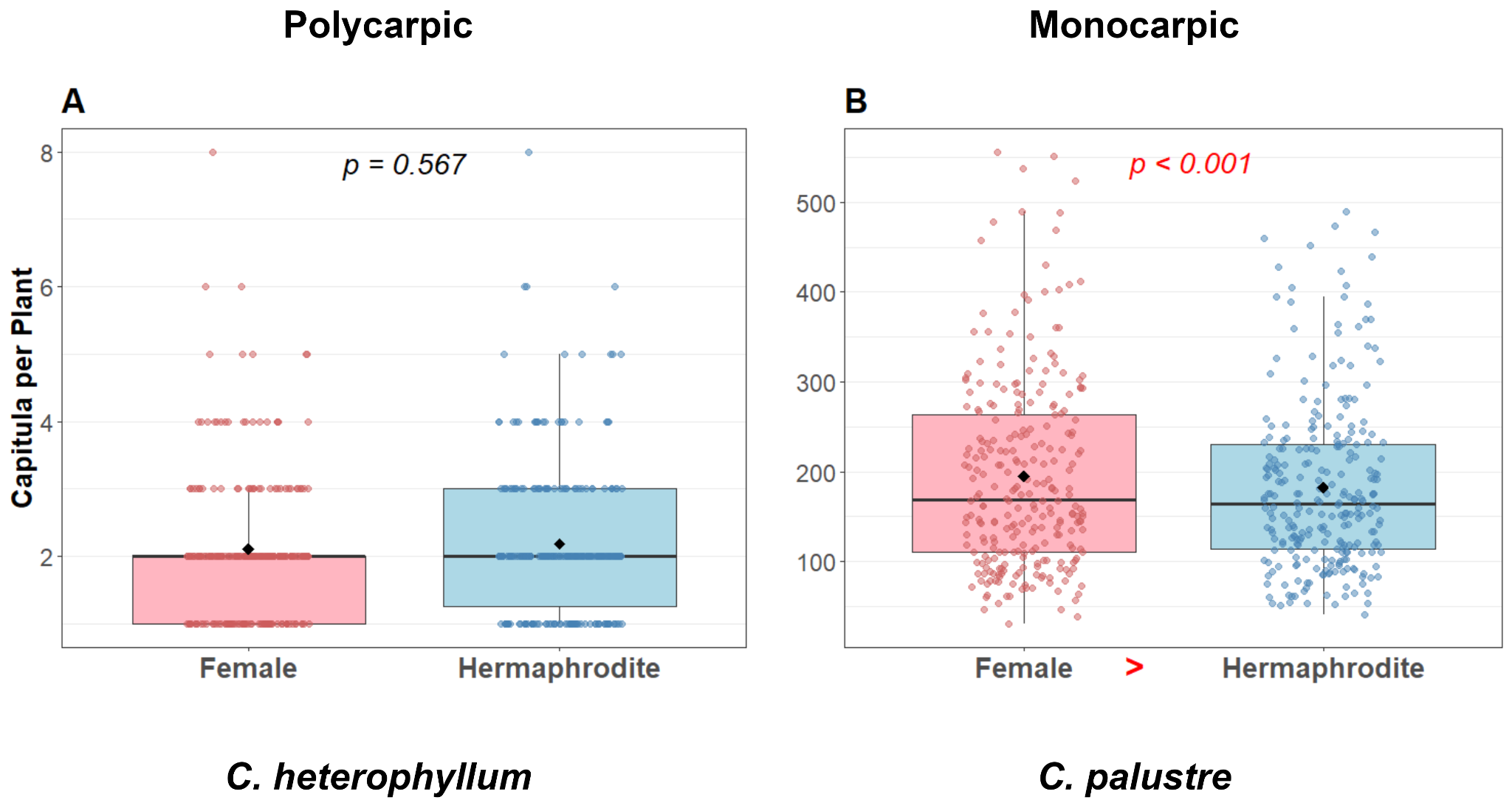
**

**Supplementary Fig. S1.** Comparison of capitula numbers per plant between female and hermaphroditic individuals in polycarpic *Cirsium heterophyllum* **(A)** and monocarpic *C. palustre* **(B)**. Significant sex differences on generalized linear mixed-effects models (GLMMs; *p* < 0.05) are shown in red, with the larger sex indicated by “>” between females (F) and hermaphrodites (H). Observed capitula per plant means (± SE) were as follows: *C. heterophyllum* — F = 2.10 ± 0.06, H = 2.18 ± 0.07 ; *C. palustre* — F = 195 ± 6.55, H = 182 ± 5.75.

**
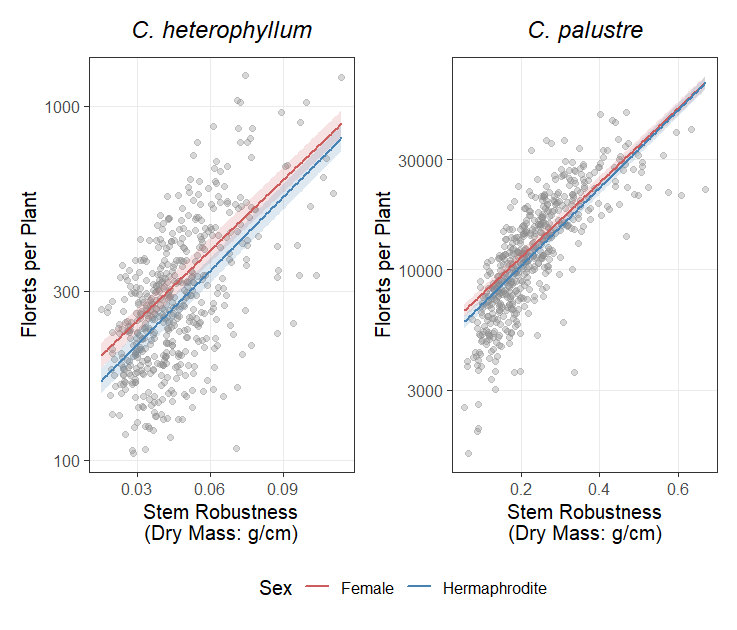
**

**Supplementary Fig. S2.** Relationship between stem robustness and florets per plant in *C. heterophyllum* and *C. palustre*. Florets per plant were analysed using Poisson generalized linear mixed-effects models (GLMMs) with locality as a random effect. For both species, the model including the Sex × Robustness interaction provided a significantly better fit than the additive model. In *C. heterophyllum*, inclusion of the interaction improved model fit (ANOVA likelihood-ratio test, LRT: χ²₁ = 7.38, *p* = 0.007; Supplementary Table 13). In *C. palustre*, florets per plant showed a highly significant Sex × Robustness interaction (ANOVA LRT: χ²₁ = 788.57, *p* < 0.001; Supplementary Table 15). Lines indicate model-predicted relationships between stem robustness and florets per plant for each sex, and shaded areas show 95% confidence intervals; grey points represent individual plants. **Note.** Across both species, florets per plant scaled positively with stem robustness, indicating that reproductive output increases with overall plant robustness at the individual level (Supplementary Tables 14 and 16). In both *C. heterophyllum* and *C. palustre*, the significant Sex × Robustness interaction demonstrates that the effect of robustness on reproductive output differs between sexes. Specifically, hermaphrodites showed a slightly stronger increase in florets with rising robustness, whereas females consistently produced more florets overall across the full robustness range.

*
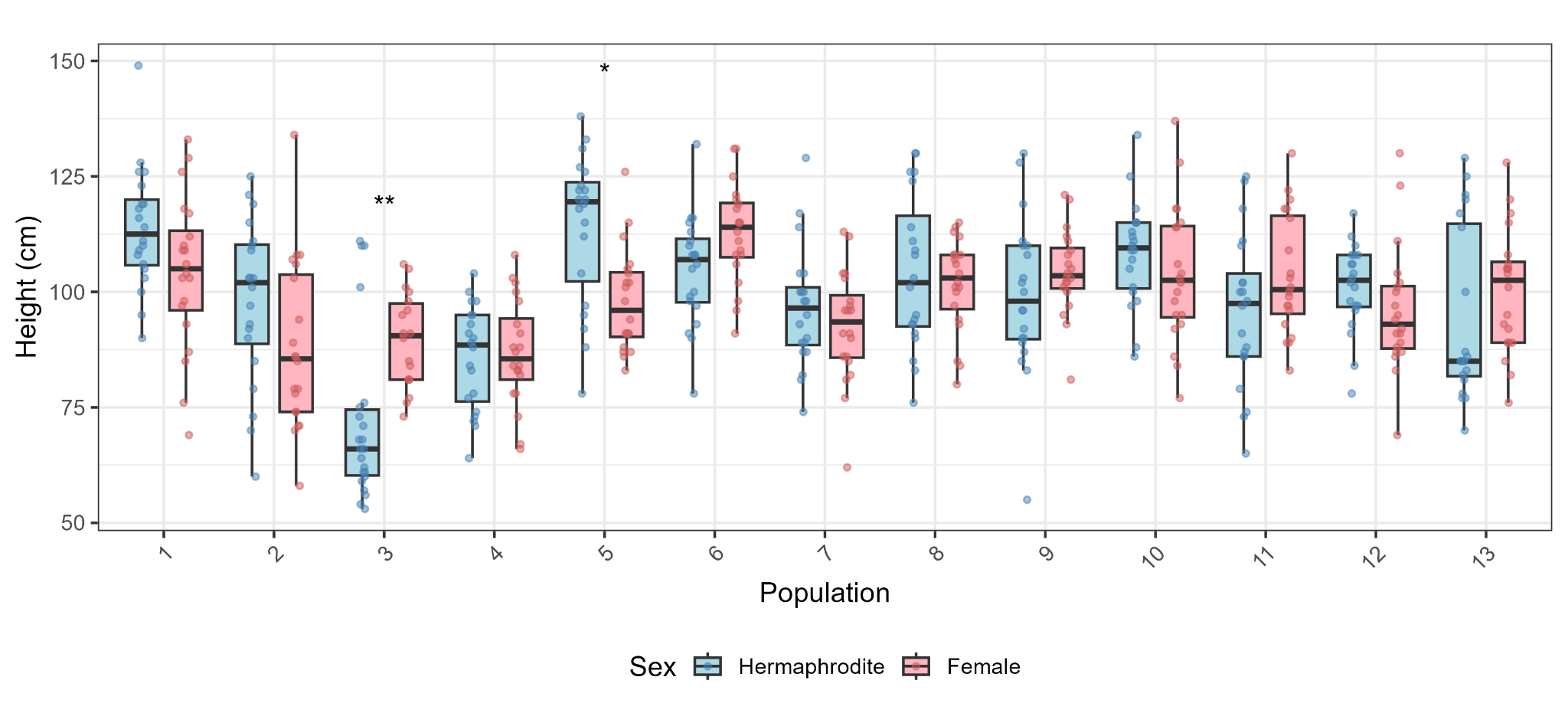
*

**Supplementary Fig. S3.** Comparison of plant height (cm) between females (red) and hermaphrodites (blue) across populations of *C. heterophyllum*. Boxplots show the interquartile range, median (thick line), and individual plants (points). Sex differences within each population were tested using Wilcoxon rank-sum tests, with significant differences indicated by asterisks (*p* < 0.05 = *; *p* < 0.01 = **). According to a two-way ANOVA, plant height differed strongly among populations ( *p* < 0.001).


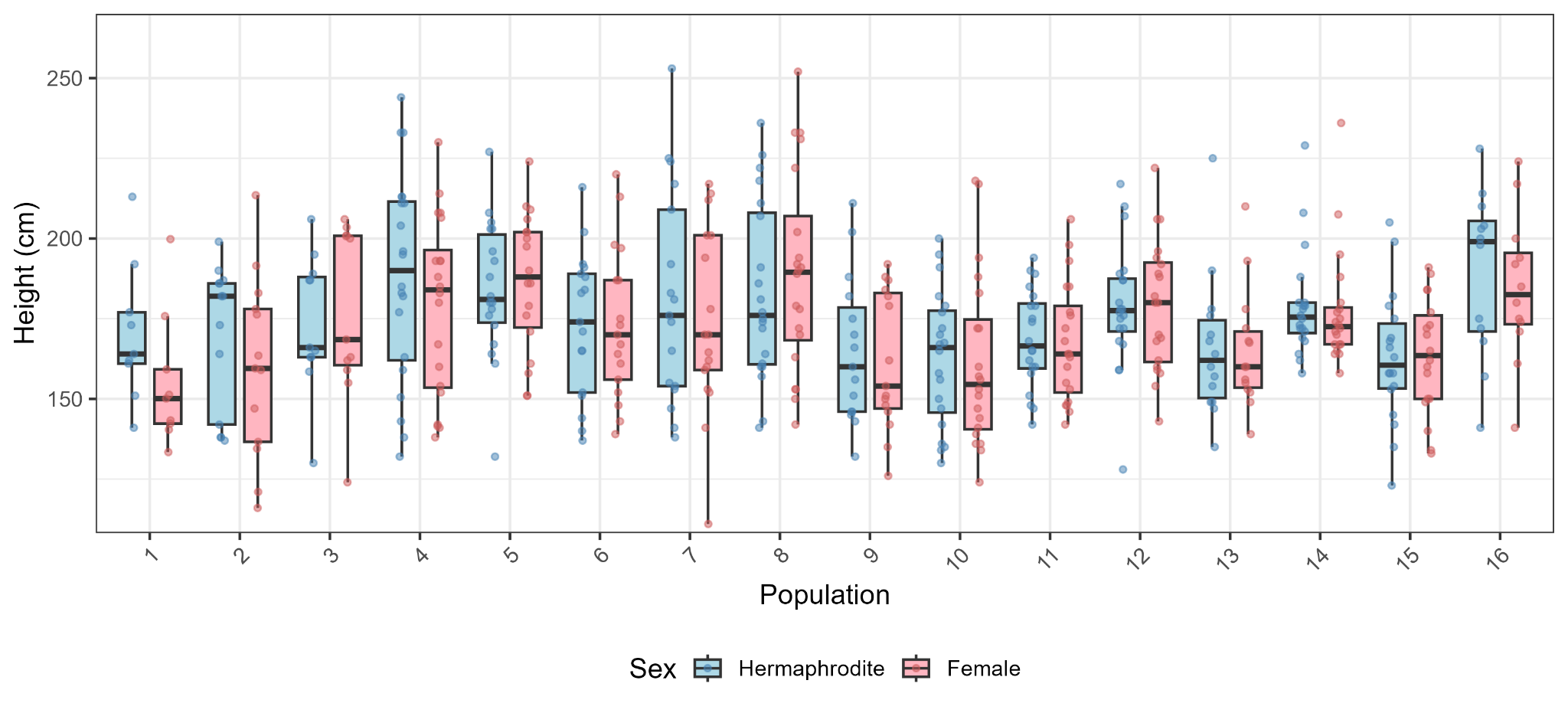


**Supplementary Fig. S4.** Comparison of plant height (cm) between females (red) and hermaphrodites (blue) across populations of *Cirsium palustre*. Boxplots show the interquartile range, median (thick line), and individual plants (points). Sex differences within each population were tested using Wilcoxon rank-sum tests, and no significant differences were detected. Based on the two-way ANOVA, plant height differed significantly among populations (*p* < 0.001).


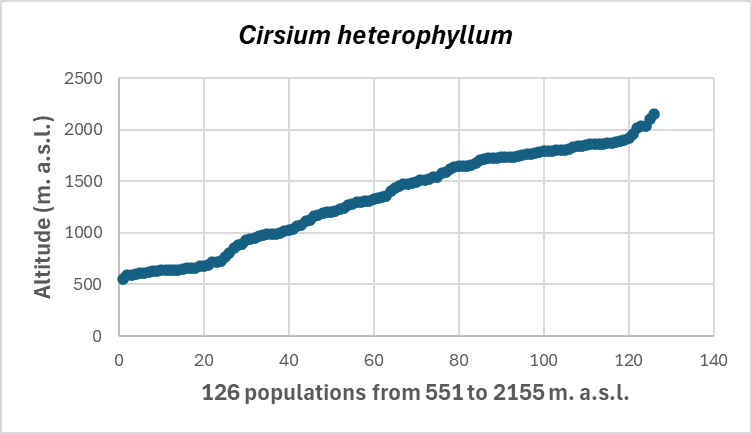


**Supplementary Fig. S5.** Altitudinal distribution of 126 populations (sample localities) of *C. heterophyllum.*


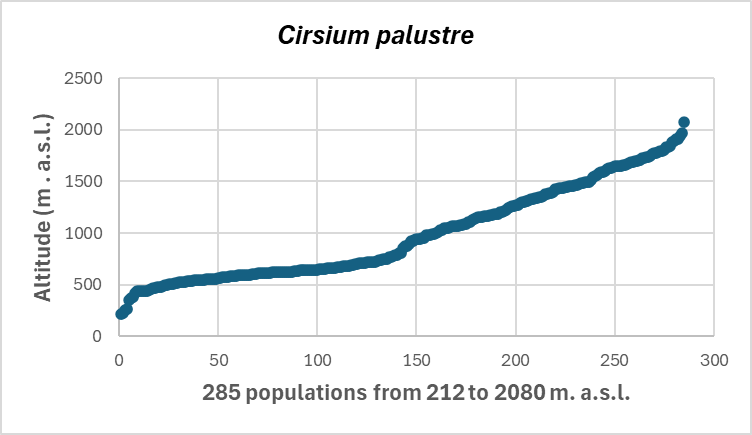


**Supplementary Fig. S6.** Altitudinal distribution of 126 populations (sample localities) of *C. palustre.*

***Environmental predictors of female frequency***

**
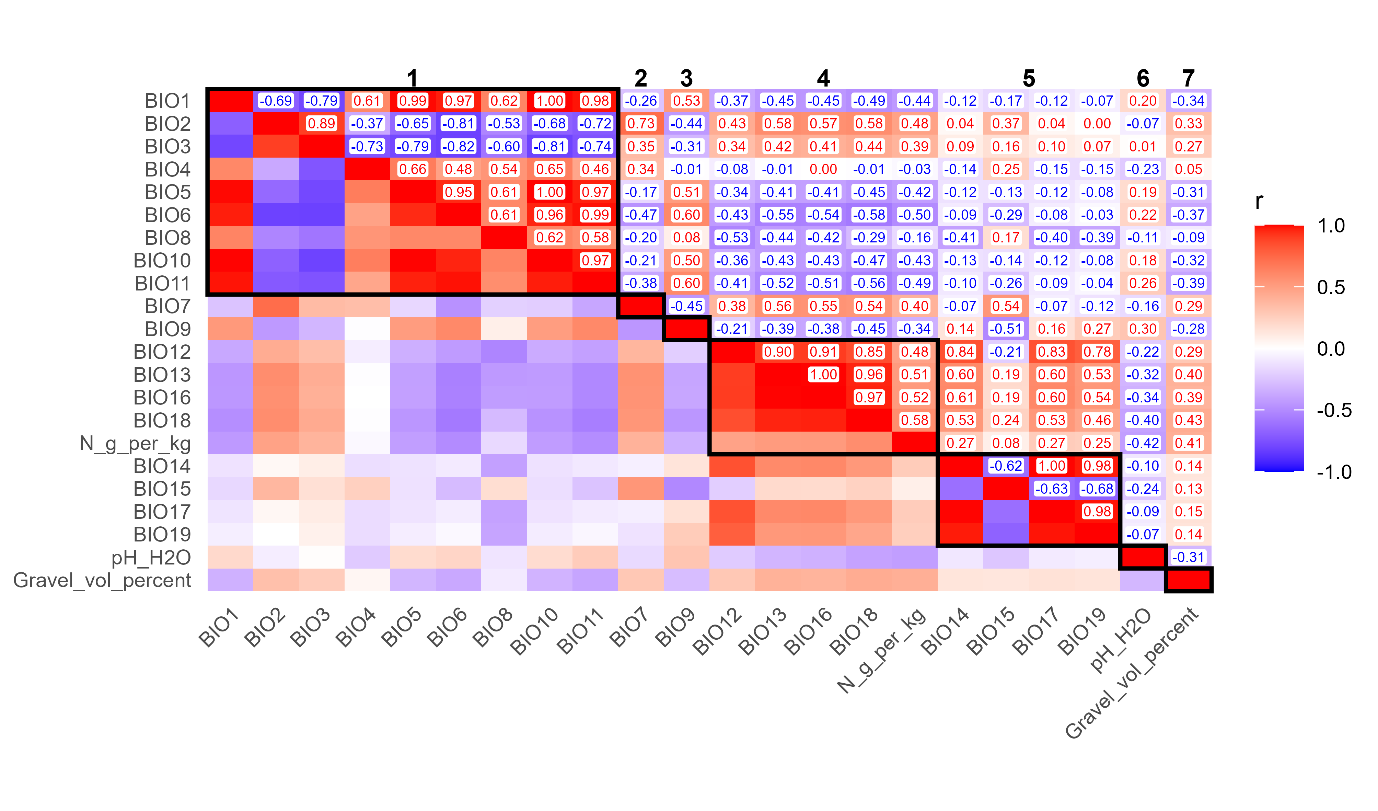
**

**Supplementary Fig. S7.** Pearson correlation heat map of the 22 environmental predictors (19 CHELSA bioclimatic variables and 3 soil variables). Variables are ordered by hierarchical clustering of |r| values, and the seven resulting predictor clusters are outlined with black rectangles. Correlation coefficients are shown in the upper triangle, while the lower triangle displays the corresponding color scale (blue = negative, red = positive). This correlation structure formed the basis of the Li & Ji (2005) spectral decomposition, yielding an effective number of independent predictors of Meff = 7.07.


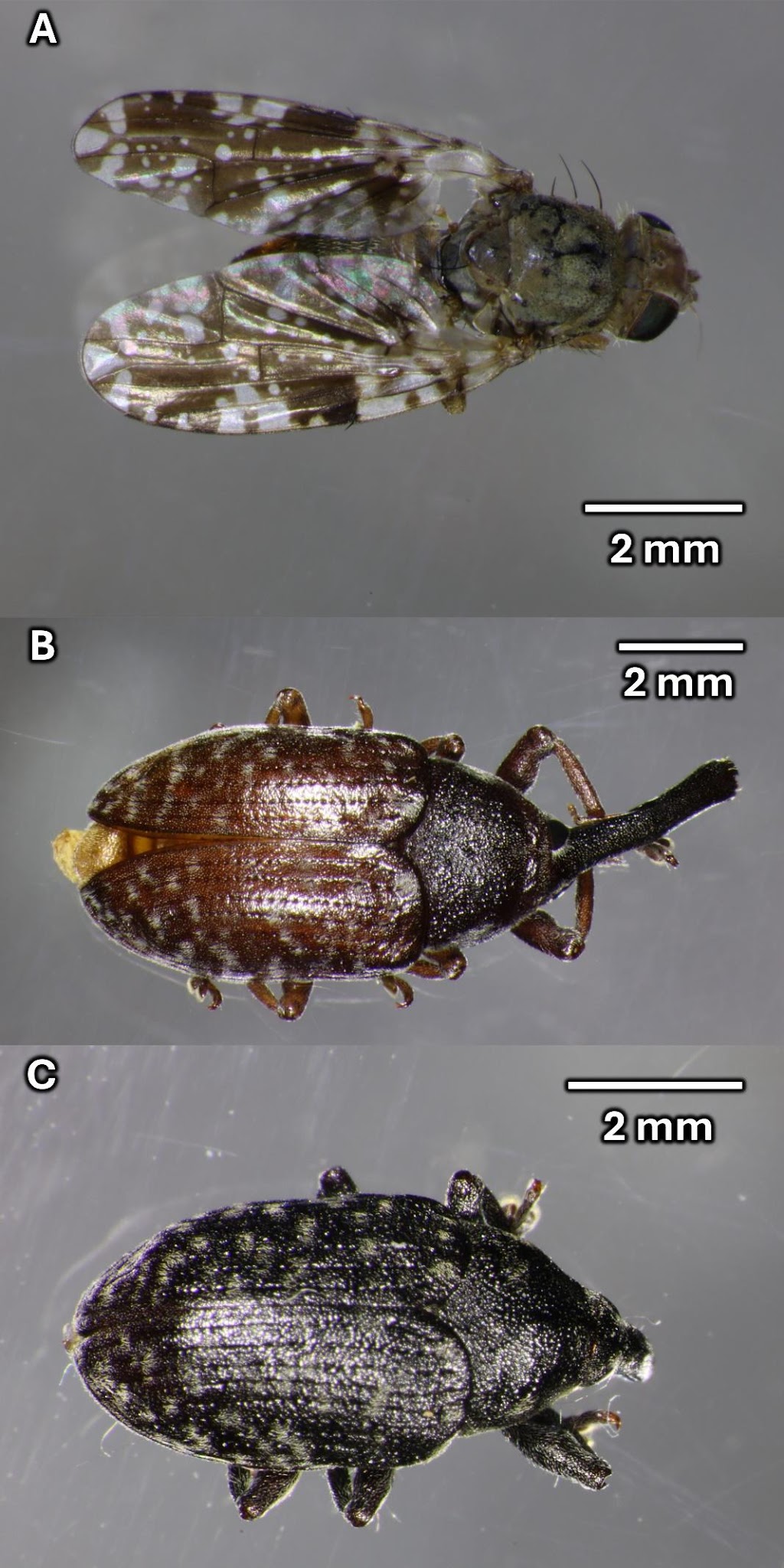


**Supplementary Fig. S8.** Identified granivores to species level. *Tephritis conura* **(A)**. *Larinus sturnus* **(B)**. *Larinus turbinatus* **(C)**. Photo credits: Petr Bureš.
